# Bacterial secretion systems contribute to rapid tissue decay in button mushroom soft rot disease

**DOI:** 10.1128/mbio.00787-23

**Published:** 2023-07-24

**Authors:** Philipp Wein, Katharina Dornblut, Sebastian Herkersdorf, Thomas Krüger, Evelyn M. Molloy, Axel A. Brakhage, Dirk Hoffmeister, Christian Hertweck

**Affiliations:** 1 Department of Biomolecular Chemistry, Leibniz Institute for Natural Product Research and Infection Biology (HKI), Jena, Germany; 2 Department of Pharmaceutical Microbiology, Leibniz Institute for Natural Product Research and Infection Biology, Friedrich Schiller University Jena, Jena, Germany; 3 Faculty of Biological Sciences, Friedrich Schiller University Jena, Jena, Germany; 4 Department of Molecular and Applied Microbiology, Leibniz Institute for Natural Product Research and Infection Biology (HKI), Jena, Germany; Karlsruhe Institute of Technology, Karlsruhe, Germany

**Keywords:** *Janthinobacterium agaricidamnosum*, lytic enzymes, mushroom pathogens, secretome analysis, type III secretion

## Abstract

The soft rot pathogen *Janthinobacterium agaricidamnosum* causes devastating damage to button mushrooms (*Agaricus bisporus*), one of the most cultivated and commercially relevant mushrooms. We previously discovered that this pathogen releases the membrane-disrupting lipopeptide jagaricin. This bacterial toxin, however, could not solely explain the rapid decay of mushroom fruiting bodies, indicating that *J. agaricidamnosum* implements a more sophisticated infection strategy. In this study, we show that secretion systems play a crucial role in soft rot disease. By mining the genome of *J. agaricidamnosum*, we identified gene clusters encoding a type I (T1SS), a type II (T2SS), a type III (T3SS), and two type VI secretion systems (T6SSs). We targeted the T2SS and T3SS for gene inactivation studies, and subsequent bioassays implicated both in soft rot disease. Furthermore, through a combination of comparative secretome analysis and activity-guided fractionation, we identified a number of secreted lytic enzymes responsible for mushroom damage. Our findings regarding the contribution of secretion systems to the disease process expand the current knowledge of bacterial soft rot pathogens and represent a significant stride toward identifying targets for their disarmament with secretion system inhibitors.

**IMPORTANCE:**

The button mushroom (*Agaricus bisporus*) is the most popular edible mushroom in the Western world. However, mushroom crops can fall victim to serious bacterial diseases that are a major threat to the mushroom industry, among them being soft rot disease caused by *Janthinobacterium agaricidamnosum*. Here, we show that the rapid dissolution of mushroom fruiting bodies after bacterial invasion is due to degradative enzymes and putative effector proteins secreted via the type II secretion system (T2SS) and the type III secretion system (T3SS), respectively. The ability to degrade mushroom tissue is significantly attenuated in secretion-deficient mutants, which establishes that secretion systems are key factors in mushroom soft rot disease. This insight is of both ecological and agricultural relevance by shedding light on the disease processes behind a pathogenic bacterial-fungal interaction which, in turn, serves as a starting point for the development of secretion system inhibitors to control disease progression.

## INTRODUCTION

The agricultural sector continually suffers severe economic losses due to the adverse effect of Gram-negative bacterial soft rot pathogens on the yields of vegetable, fruit, and mushroom farming (1 - 4). These bacteria exert their devastating effects by releasing multiple virulence factors, lytic enzymes, and toxins to exploit eukaryotic host cells, thereby degrading the crop to mush (5 - 7). While the disease processes of plant-infecting soft rot pathogens are well studied, with many using dedicated secretion systems to deliver disease mediators, their mushroom-infecting counterparts are poorly understood (8, 9). Indeed, *Janthinobacterium agaricidamnosum* DSM 9628 is a causative agent of mushroom soft rot disease, but the associated virulence determinants remain obscure. *J. agaricidamnosum* infection of the button mushroom *Agaricus bisporus* is characterized by pitting, sticky blotches, and rapid dissolution of fruiting bodies (10). Awareness of this pathogen is of particular importance to mushroom farming since *A. bisporus* is one of the most cultivated and widely consumed mushrooms, constituting around 15% of the world’s supply (11). Intriguingly, the *Janthinobacterium* genus is considered non-pathogenic, with members being commonly isolated from water, forest soils, Arctic, and Antarctic habitats (12 - 16). Besides *J. agaricidamnosum*, only very few representatives of the genus engage in a pathogenic lifestyle (17, 18), among them *Janthinobacterium lividum* has been associated with mortal disease of rainbow trouts and human sepsis (19 - 21). Conversely, *J. lividum* is also known as cutaneous mutualistic bacterium that protects amphibians from the lethal skin disease chytridiomycosis (22, 23). The protective effect has been traced to the antifungal secondary metabolite violacein, whose intense purple color is eponymous for the genus *Janthinobacterium* (23, 24). We previously implicated another antifungal secondary metabolite, the cyclic lipopeptide jagaricin, in the mushroom soft rot disease caused by *J. agaricidamnosum* (25). This toxin acts by impairing the membrane integrity of host cells, resulting in a rapid influx of ions, including Ca^2+^ (26). However, the striking soft rot symptoms could not be explained solely by the lipopeptide (25). Thus, the molecular basis of this mushroom soft rot disease and the effectors involved remained elusive. Here, we show that the type II secretion system (T2SS) and type III secretion system (T3SS) of *J. agaricidamnosum* are central to its infection strategy and implicate a number of T2SS-secreted lytic enzymes in the deleterious effects on *A. bisporus*. Providing detailed knowledge on the pathobiology of soft rot pathogens may aid the development of protective measures against devastating losses in mushroom agriculture.

## RESULTS AND DISCUSSION

### *J. agaricidamnosum* harbors four different secretion systems

**Fig 1:**
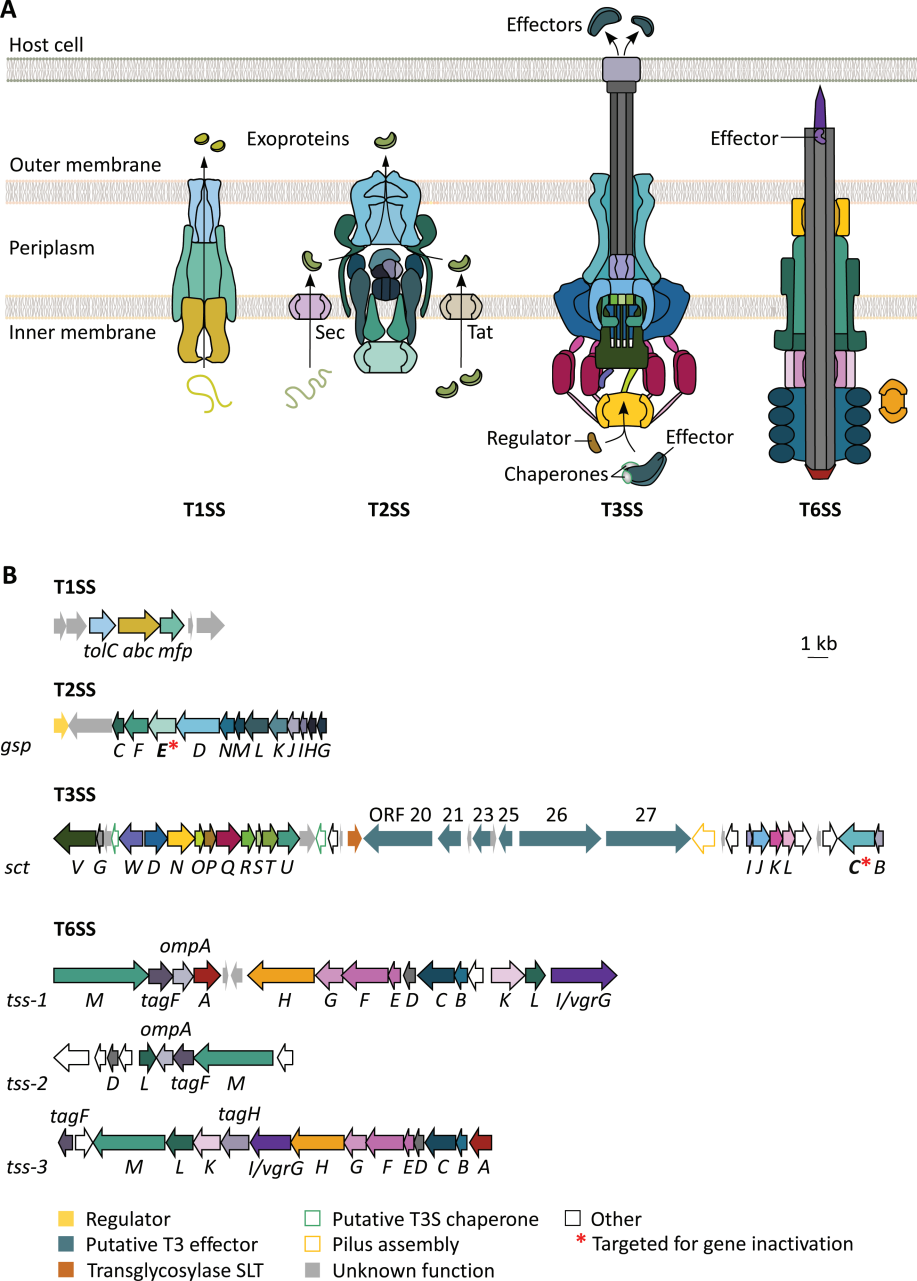
The *Janthinobacterium agaricidamnosum* genome encodes four different secretion systems. (**A**) Schematic representation of the type 1, 2, 3, and 6 secretion systems (T1SS, T2SS, T3SS, and T6SS) of *J. agaricidamnosum*. (**B**) Genetic organization of the T1SS, T2SS (*gsp*), T3SS (*sct*), and T6SS (*tss*) gene clusters within the genome of *J. agaricidamnosum*. Arrows display protein-coding regions. Conserved genes encoding secretion system core proteins with similarity to well-characterized secretion system components are represented by arrows with letter designations (color code continues from panel A). The predicted functions of other gene products are indicated in the key. T3 secretion signals of open reading frames (ORFs) 20, 21, 23, and 25–27 were predicted with EFFECTIVE T3 software.

Given that some of the most virulent Gram-negative bacteria use secretion systems to release or inject toxic proteins into eukaryotic host cells (27, 28), we browsed the whole-genome sequence of *J. agaricidamnosum* (GenBank accession number: HG322949) for potential secretion system gene clusters. In doing so, we uncovered four different types that were scattered separately throughout the genome (Fig. 1A and B). The products of unannotated genes encoded within the putative clusters were further inspected for their similarity to characterized proteins using the basic local alignment search tool (BLAST) (29) and HHpred (30) (see Table S1 in the supplemental material).

We found three genes that together potentially encode a type I secretion system (T1SS). In pathogenic bacteria, these systems often secrete protein substrates implicated in virulence and nutrient acquisition (31 - 34). Furthermore, we identified a gene cluster coding for 12 proteins of the general secretion pathway (GspC–N), suggesting that the bacterium has a functional T2SS. We also discovered a large T3SS gene cluster that spans about 42,000 bp and contains 39 open reading frames (ORFs). A protein BLAST search using the well-conserved SctC (secretin) and SctT (export apparatus) revealed that among the 106 publicly available *Janthinobacterium* whole-genome sequences, only the environmental isolates *Janthinobacterium* sp. B9-8 and the red-pigmented *Janthinobacterium* sp. BJB412 also harbor genes for a functional T3SS (see Fig. S1 in the supplemental material). Six ORFs (ORF 20, 21, 23, and 25–27) within the T3SS cluster of *J. agaricidamnosum* encode proteins with unknown functions that possess N-terminal T3SS signals as predicted by the EFFECTIVE T3 tool (35), indicating that these proteins might be T3 effectors (Table S2). ORF 20 (GJA_RS07825) codes for a protein that exhibits similarity (24%) to the alanine-tryptophan-arginine triad (AWR, also named RipA) effector family of the plant pathogen *Ralstonia solanacearum* (36). Although their putative function cannot be deduced from the protein sequence information alone, it has been shown that AWR T3 effectors contribute to *R. solanacearum* virulence in numerous ways (37). A multiple sequence alignment and phylogenetic analyses demonstrated that the AWR effector of *J. agaricidamnosum* deviates from the AWR members of *R. solanacearum* in regard to the conserved regions (data set Fig. S2 to S4). The putative effector encoded by ORF 21 (GJA_RS07835) does not share similarity with any known protein. The ORFs 23 (GJA_RS27170) and 25–27 (GJA_RS07855, GJA_RS07860, and GJA_RS07865) encode proteins that exhibit sequence similarities (26%–37%) to putative T3 effectors with unknown function from various plant pathogens, including *Pseudomonas* spp., *Xanthomonas* spp., and *R. solanacearum*. In addition, we encountered two complete gene clusters (*tss*-1 and *tss*-3) and an incomplete cluster (*tss*-2) that are reminiscent of type VI secretion system (T6SS) clusters. The effectors delivered by this type of contractile nanomachine usually have a pivotal role not only in interbacterial and interkingdom competition (38 - 40) but also in bacterial-host interactions and nutrient acquisition (41 - 44).

Any of the detected putative secretion systems (T1SS, T2SS, T3SS, and T6SSs), or a combination thereof, may be involved in the interaction of *J. agaricidamnosum* with *A. bisporus*. Because of the known degradative nature of T2SS-dependent proteins (45), we deemed the bioinformatically identified T2SS a likely candidate to promote the damage of mushroom tissue. In addition, considering the role of T3SS-associated effectors in human pathogens like *Salmonella* or *Yersinia* (46), and plant pathogens like *Pseudomonas syringae* and *R. solanacearum* (47, 48), we wondered whether the predicted T3SS might be also involved in mushroom soft rot.

### Deficiency in T2SS or T3SS attenuates the capacity of *J. agaricidamnosum* to cause button mushroom soft rot

**Fig 2:**
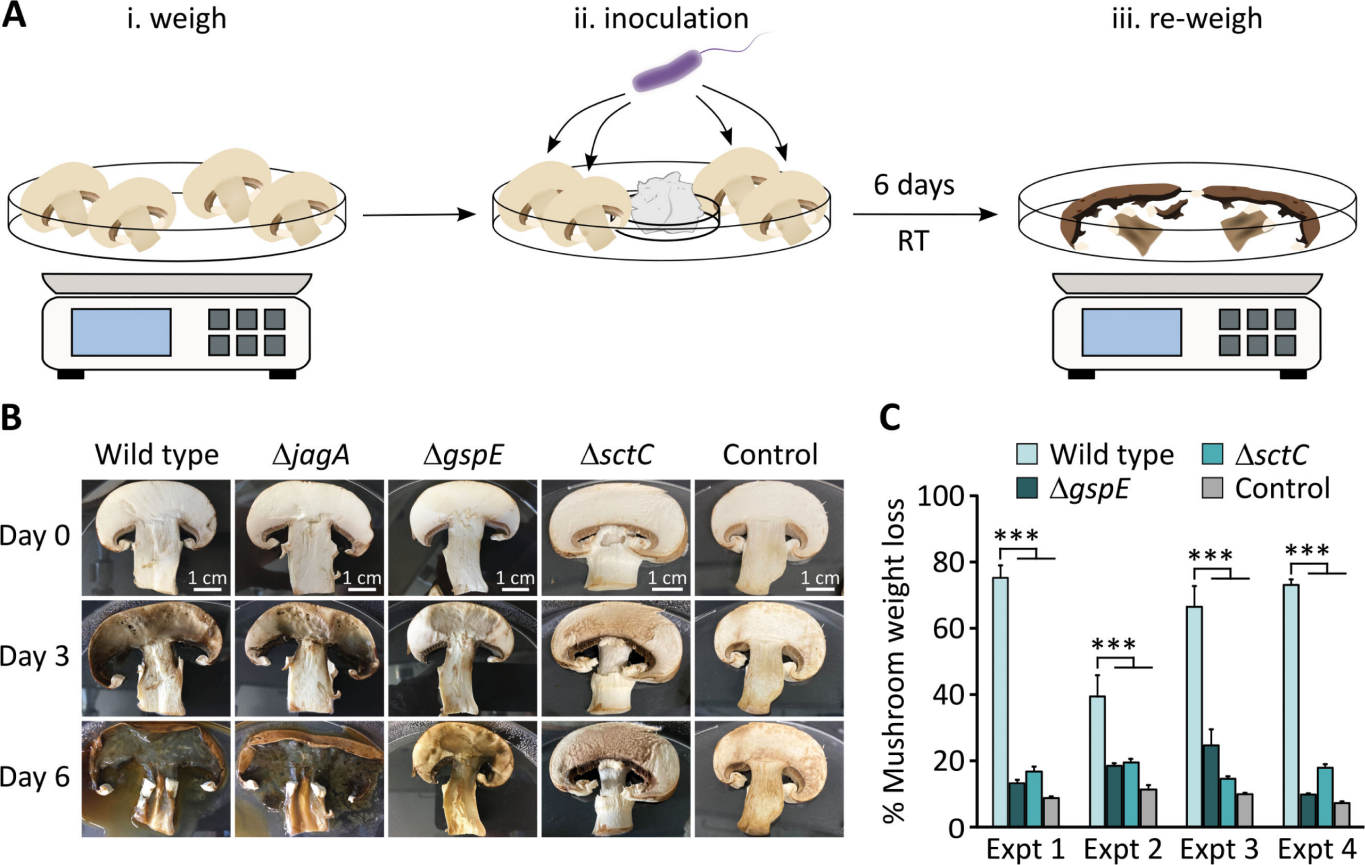
Contribution of T2SS and T3SS to soft rot disease. (**A**) Schematic illustration of the button mushroom infection assay. Mushroom slices were placed in a petri dish and weighed (i), then inoculated with 2.7 × 10^7^ cells of *J. agaricidamnosum* or mutants onto two different spots on each trama (ii). A wet sterilized tissue was placed in the center of the petri dish to prevent the slices from drying out. After 6 days of incubation, non-degraded mushroom tissue was collected and re-weighed to determine the level of degradation (iii). RT, room temperature. (**B**) Representative images of mushroom slices infected with *J. agaricidamnosum*, *J. agaricidamnosum* Δ*jag*, *J. agaricidamnosum* Δ*gspE,* or *J. agaricidamnosum* Δ*sctC* from day 0 to day 6. (**C**) Statistical evaluation of infected mushroom slices after 6 days of inoculation. Mushrooms slices inoculated with *J. agaricidamnosum* Δ*gspE*, *J. agaricidamnosum* Δ*sctC,* or culture medium (control) show significantly less (*P* < 0.05) degrees of degradation when compared to mushrooms slices infected with *J. agaricidamnosum* wild type. The results depicted are derived from four independent experiments. The values displayed for each experiment are the mean of six replicates consisting of four mushroom slices each, with error bars showing the standard error of the mean. ***P < 0.001 (Bonferroni’s means comparison test, two-way analysis of variance [ANOVA]).

In order to investigate whether the bioinformatically identified T2SS and T3SS of *J. agaricidamnosum* play a role in pathogenesis, we first verified by reverse-transcriptase PCR that the associated genes (*gspD*, *gspE*, *sctC*, and *sctT*) are expressed under laboratory conditions (Fig. S5). Subsequently, we inactivated the relevant genetic loci. To this end, we targeted the secretin encoding gene *gspD* as well as *gspE*, which encodes an ATPase, the driving force of the T2SS machinery. In case of the T3SS, the secretin-encoding gene *sctC* and the inner membrane export apparatus protein encoded by *sctT* were targeted. Due to the lack of genetic tools and standard cloning procedures for *J. agaricidamnosum*, we adapted a double-crossover strategy, previously applied to identify the biosynthetic gene cluster of jagaricin (25), to disrupt the corresponding genes with a kanamycin resistance cassette. We succeeded in generating one of the two desired mutations to each secretion system, resulting in the T2SS-deficient strain *J. agaricidamnosum* Δ*gspE* and the T3SS-deficient strain *J. agaricidamnosum* Δ*sctC* (Fig. S6 and S7). Both mutants do not show morphological differences or defects in growth and motility when compared to the wild type under standard laboratory conditions (Fig. S8 and S9). We proceeded to examine the effect of the mutations on pathogenesis by inoculating the trama (inner flesh of the fruiting body) of separate button mushroom slices with *J. agaricidamnosum*, *J. agaricidamnosum* Δ*gspE*, and *J. agaricidamnosum* Δ*sctC* (Fig. 2A). We also included the jagaricin-deficient strain *J. agaricidamnosum* Δ*jagA* (25) for comparison. First, a visual inspection of severity of soft rot symptoms was performed (Fig. 2B). Subsequently, we also quantified the level of mushroom degradation by comparing the weight of starting mushroom tissue with that of degraded tissue after 6 days in four independent experiments (Fig. 2C). We found that the T2SS- and T3SS-deficient mutants cause significantly reduced trama degradation. While *J. agaricidamnosum* lysed the mushroom tissue almost completely, the infection with *J. agaricidamnosum* Δ*gspE* and *J. agaricidamnosum* Δ*sctC* led to less severe symptoms such as browning and sticky blotches. We noted that after 3 days of infection, *J. agaricidamnosum* wild type, and to a lesser extent *J. agaricidamnosum* Δ*gspE,* cause characteristic cavities on the mushroom slices. In contrast, these cavities are absent on mushroom slices infected with *J. agaricidamnosum* Δ*sctC*. Furthermore, the antifungal effect of jagaricin is not apparent in this mushroom infection assay since there was no visible difference in symptoms caused by *J. agaricidamnosum* Δ*jagA* compared to the *J. agaricidamnosum* wild type, corroborating that additional factors contribute to the infection strategy (25).

This finding that secretion systems are involved in mushroom soft rot disease is interesting in light of known pathomechanisms of soft rot pathogens. For instance, the pathogenic strategies of fruit- and vegetable-infecting *Dickeya* spp. and *Pectobacterium* spp. require a T2SS and its secreted proteins (5, 49). While T3SS-secreted effector proteins make only a small contribution to the pathogenicity of *Pectobacterium* spp. (50), they play a key role in the initial infection process of *Dickeya dadantii* (51). However, little is known about the role of T3SS in bacterial-fungal interactions, whether they be mutualistic or pathogenic in nature (52, 53). Only a few studies have been reported showing that T3SSs contribute to pathogenic bacterial-fungal interactions (54, 55). For example, *Salmonella enterica* serovar Typhimurium competes with the pathogen *Candida albicans* by releasing an inositol phosphatase (SopB) via a T3SS that negatively affects the viability of the fungus (55). The bioinformatically identified T3 effector proteins of *J. agaricidamnosum* exhibit low similarity to phytopathogenic effectors, some of which are known to alter plant physiology and interfere with the plant immune response (47). However, similar functions cannot simply be predicted since those processes are unknown in higher fungi like *A. bisporus*. Nonetheless, the mushroom infection assays indicated that both T2SS- and T3SS-secreted proteins are involved in mushroom trama damage.

### The T2SS releases tissue-degrading and chitinolytic proteins

**Fig 3:**
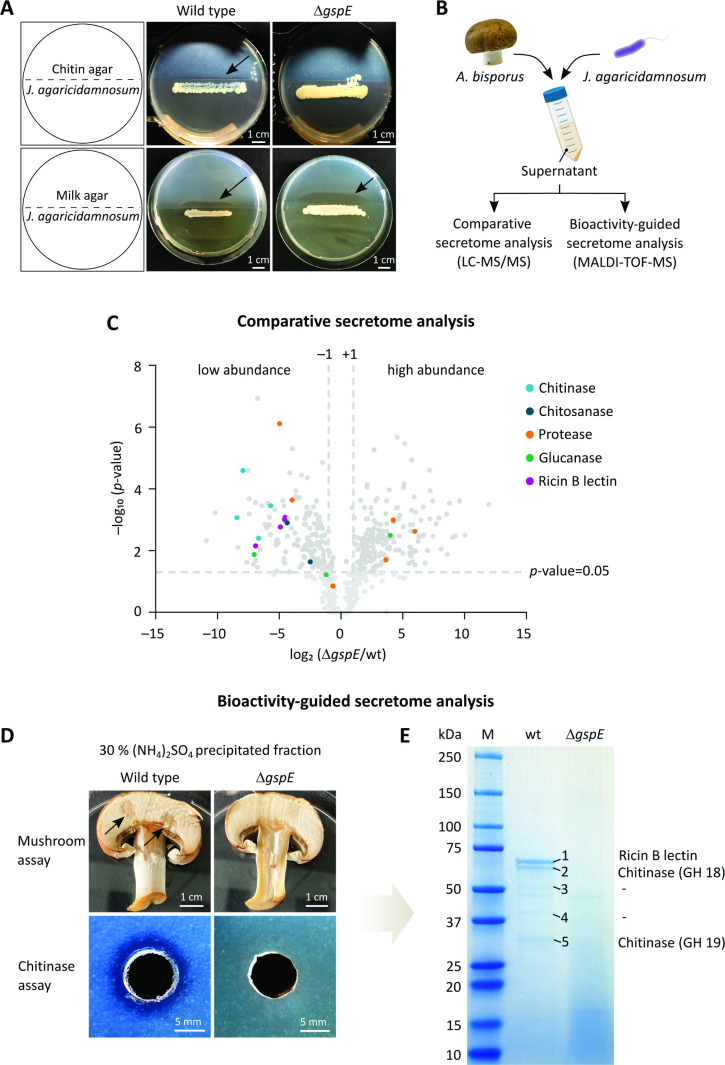
Bioactivity-guided secretome analysis. (**A**) Split-plate assay with chitin (top row) or milk agar (bottom row) to screen for secreted chitin- and protein-degrading enzymes of *J. agaricidamnosum* (wild type) and T2SS-deficient *J. agaricidamnosum* Δ*gspE*. Halo formation indicates protease activity from *J. agaricidamnosum* and *J. agaricidamnosum* Δ*gspE*. Chitinase activity was only observed from *J. agaricidamnosum* as indicated by a halo. Arrows point to visible halos caused by enzymatic activity. (**B**) Workflow for the secretome analysis of *J. agaricidamnosum* or *J. agaricidamnosum* Δ*gspE* cultivated in media containing button mushrooms as the sole nutrient source. (**C**) Volcano-plot visualization of protein abundance changes [threshold of log_2_(Δ*gspE*/wt) < −1 and >1 and the ratio adjusted *P*-value < 0.05] obtained from comparative LC-MS/MS secretome analysis of *J. agaricidamnosum* and *J. agaricidamnosum* Δ*gspE*. The significantly lower abundance of lytic enzymes (chitinases, chitosanases, proteases, glucanases, and ricin B lectins) in the *J. agaricidamnosum*Δ*gspE* secretome indicates their impaired secretion in the absence of a functional T2SS. (**D**) Bioactivity-guided secretome analysis of *J. agaricidamnosum* and *J. agaricidamnosum* Δ*gspE* reveals that a single protein fraction [precipitated in 30% (NH_4_)_2_SO_4_ saturation] from *J. agaricidamnosum* causes lesions on mushroom slices and halo formation on chitin agar. Arrows point to visible lesions. (**E**) MALDI-TOF-MS of SDS-PAGE-separated proteins unveil two chitinases of the glycoside hydrolase family 18 and 19 and a ricin-type beta-trefoil lectin domain protein in the bioactive fraction.

Based on the observation that both the T2SS- and the T3SS-deficient mutants are impaired in their ability to cause soft rot disease symptoms, we assumed the lack of degradative proteins may explain this phenotype. Whereas putative T3 effector proteins are encoded within the T3SS gene cluster, the genomic neighborhood of the T2SS gene cluster does not reveal candidates that could contribute to mushroom soft rot disease. However, T2SSs are typically involved in secreting toxins and lytic enzymes (45). An extensive *in silico* secretome analysis confirmed that the *J. agaricidamnosum* genome contains a number of genes that encode putative exoproteins with N-terminal secretion signals such as lytic enzymes (data not shown). We monitored *J. agaricidamnosum* and *J. agaricidamnosum* Δ*gspE* for differences in chitinolytic and proteolytic activity by culturing them on split plates containing standard growth agar on one half of the petri dish and chitin or milk agar on the other. Proteolytic activity was not visibly affected by the inactivation of the T2SS; both strains showed strong proteolytic activity on milk agar. While *J. agaricidamnosum* displayed chitinolytic activity, visible by a halo on the chitin agar, chitin degradation was not detected for *J. agaricidamnosum* Δ*gspE* (Fig. 3A).

Intrigued by the observation that the secretion of chitinases is impaired in the absence of a functional T2SS, we set out to identify the T2SS-secreted suite of chitinases and other proteins. Therefore, we performed a comparative analysis of the total secretomes of *J. agaricidamnosum* and *J. agaricidamnosum* Δ*gspE*. Both strains were cultured in medium containing lyophilized, ground button mushrooms as the sole nutrient source (Fig. 3B). Subsequently, the secretomes were analyzed by means of LC-MS/MS, demonstrating that two proteases and one glucanase are less abundant in the secretome of *J. agaricidamnosum* Δ*gspE* compared to that of *J. agaricidamnosum* wild type (Fig. 3C). Surprisingly, three proteases and another glucanase are more abundant in the *J. agaricidamnosum* Δ*gspE* secretome. This finding is consistent with the observation that both *J. agaricidamnosum* and *J. agaricidamnosum* Δ*gspE* show protease activity on milk agar, indicating that certain exoproteins can be secreted independently of the T2SS. The production of exoproteins, such as extracellular proteases, is known to be regulated by the repression of carbon, nitrogen, and sulfur in bacteria (56). Considering that the T2SS-deficient mutant has diminished capacity to secrete various hydrolyzing enzymes that provide utilizable breakdown products needed for growth and survival, it is conceivable that the bacterium responds with increased exoprotein production. Thus, it could be that proteins that are secreted independently of the T2SS appear more abundant in the supernatant of the T2SS-deficient mutant.

Notably, the triplicate experiments demonstrated that chitinases and chitosanases are substantially less abundant in the secretome of the T2SS-deficient mutant in agreement with the phenotypes observed in the chitinase activity assay (Fig. 3A and C). Moreover, while we did not detect T3 effector proteins in the supernatants of *J. agaricidamnosum* and *J. agaricidamnosum* Δ*gspE*, the secretion of proteins related to glucanases and ricin-type β-trefoil lectin (RICIN) domain-containing proteins appears to be impaired in the T2SS-deficient mutant (Fig. 3C; Table 1).

**TABLE 1:** Proteins identified by LC-MS/MS that are predicted as exoproteins^*a*^

NCBI accession no.	Protein identification	Pfam	Mass (kDa)	Log_2_ ratio Δ*gspE*/wild type	Signal peptide
CDG84650	Chitinase (GH 18)^*b*^	PF00704	47.2	−8.419	Yes
CDG84085	Chitinase (GH 18)	PF00704	59.8	−7.942	Yes
CDG85585	Glucanase (GH 8)	PF01270	46.4	−7.028	Yes
CDG86073	Ricin B lectin	PF14200	12.7	−6.902	Yes
CDG82113	Chitodextrinase (GH 18)	PF00704	83.8	−6.675	Yes
CDG81136	Chitinase (GH 19)	PF00182	29.0	−5.700	No^*c*^
CDG82784	Ricin B lectin	PF14200	77.8	−4.904	Yes
CDG84529	Leucyl aminopeptidase	PF04389	55.6	−4.982	Yes
CDG83702	Ricin B lectin (GH 71/99)	PF14200	51.9	−4.568	No^*c*^
CDG86074	Ricin B lectin (GH 71/99)	PF14200	51.8	−4.528	No^*c*^
CDG81767	Chitosanase (GH 46)	PF01374	30.9	−4.356	Yes
CDG85621	Peptidase S41 family protein	PF00595	53.5	−3.958	Yes
CDG81764	Chitosanase (GH 46)	PF01374	28.7	−2.490	Yes
CDG84341	Endo-1,3(4)-β-glucanase (GH 81)	PF00754	136.0	−1.208	Yes
CDG84587	Subtilisin-like protease	PF00082	106.3	−0.662	Yes
CDG82666	Peptidase M28 family protein	PF04389	50.7	3.629	Yes
CDG82433	Endoglucanase (GH 9)	PF00759	64.9	3.978	Yes
CDG85746	Peptidase M13 family protein	PF01431, PF05649	76.0	4.223	Yes
CDG85353	Peptidase M28 family protein	PF04389	58.8	5.978	Yes

^*a*^ Secretion signal peptide (SP) was predicted with SignalP 6.

^*b*^ GH, glycoside hydrolase family protein.

^*c*^ Non-classical secretion was predicted with SecretomeP 2.0.

To identify the exoproteins responsible for soft rot symptoms, we again cultured *J. agaricidamnosum* and *J. agaricidamnosum* Δ*gspE* in button mushroom media. The secretomes were fractionated by ammonium sulfate precipitation of filtered supernatants and, after dialysis, tested in a mushroom assay and on chitin agar. Whereas protein fractions of *J. agaricidamnosum* Δ*gspE* sporadically elicited mushroom browning where spotted, one particular fraction of *J. agaricidamnosum* [30% (NH_4_)_2_SO_4_ saturation] repeatedly showed chitinolytic activity and caused superficial lesions on mushroom tissue (Fig. 3D; Fig. S10). Importantly, the equivalent fractions from the T2SS-deficient mutant were inactive. When heat-treated aliquots of the *J. agaricidamnosum* active fraction were tested in the mushroom assay and on chitin agar, neither activity was detected. Protein separation of all fractions by SDS-PAGE revealed five visible protein bands between 30 and 70 kDa in the active fraction that were not present in any fractions of *J. agaricidamnosum* Δ*gspE* (Fig. 3E; Fig. S11). Due to low abundance, the two proteins of approximately 40 and 50 kDa in size could not be identified. However, an analysis of the remaining proteins by MALDI-time of flight mass spectrometry (MALDI-TOF-MS) led to the identification of three proteins, each possessing signal sequences for secretion (Table S3), that are encoded in the *J. agaricidamnosum* genome (Fig. S12 to S16). The largest protein (approximately 70 kDa) is predicted to comprise an N-terminal RICIN domain and a C-terminal right-handed β-helix structure that is common to proteins belonging to the pectate lyase superfamily (Fig. S12A through C) (57). Pectolytic enzymes like pectin lyases and pectate lyases cleave pectin, a major component of the cell wall of higher plants, and play important roles as virulence factors of plant pathogens such as *D. dadantii* and *Pectobacterium carotovorum* (49, 58). Several pectate lyases of these bacteria are known to be secreted by the T2SS. However, these enzymes feature an N-terminal fibronectin type III fold (Fn3) domain (59), rather than the RICIN domain observed here. Notably, the N-terminal domain determines the mode of secretion. Nevertheless, since the fungal cell wall does not contain pectin or pectate, the mechanism by which the predicted pectin lyase could contribute to *J. agaricidamnosum* pathogenicity is not obvious (60). An analysis of structural homology using HHpred indicates that the RICIN domain-containing protein shares more similarity with characterized exo-1,3-β-glucanases (*E*-value 3.4E^−91^, 54% identity) than with pectate lyases (*E*-value 1.6E^−5^, 11% identity) (Table S4). The two proteins of approximate sizes 60 and 30 kDa could be assigned as chitinases that belong to the glycoside hydrolase families 18 and 19, respectively (Fig. 3E, and data set Fig. S13 and S16). One could envision that glucanases and chitinases degrade mushroom tissue since glucan and chitin are major components of the *A. bisporus* cell wall, along with other polysaccharides such as mannan and chitosan (60). This enzymatic activity could potentially facilitate access of jagaricin to its target, the cell membrane. An analogous case in which both T2SS-secreted chitinases and secondary metabolites support the infection process has been described for the mushroom pathogen *Burkholderia gladioli* pv. *agaricicola* (7, 61, 62). Considering the variety of lytic enzymes identified in the secretome of *J. agaricidamnosum*, we assume that *J. agaricidamnosum* releases an armory of lytic enzymes to decompose mushroom tissue.

**Fig 4:**
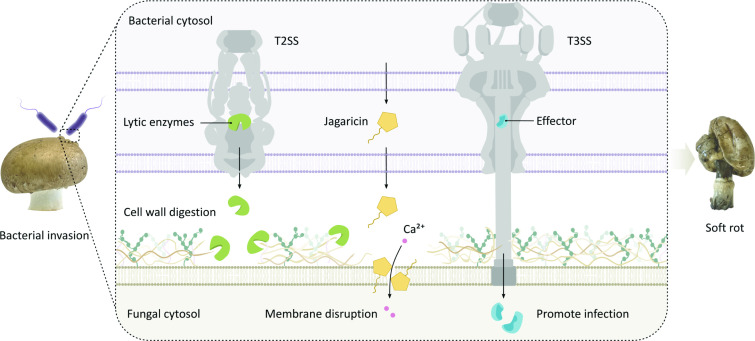
Model of the pathogenic mechanism of *J. agaricidamnosum* involved in button mushroom soft rot disease. Lytic enzymes secreted via a bacterial T2SS induce the decay of the *A. bisporus* cell wall. This enables virulence factors such as jagaricin to attack the fungal membrane. T3 effector proteins are secreted directly into the host cell and promote the infection process by a so far unknown mechanism.

In conclusion, we demonstrated that various factors contribute to the pathogenicity of *J. agaricidamnosum*. We successfully constructed mutant strains that enabled the role of the T2SS and T3SS in pathogenesis to be dissected. Given the stark differences between the phenotypes of *J. agaricidamnosum* and the secretion system deficient mutants *J. agaricidamnosum* Δ*gspE* and *J. agaricidamnosum* Δ*sctC* in the mushroom infection assay, we implicate T2SS- and T3SS-secreted proteins of *J. agaricidamnos*um in the damage of its fungal host. The collective results of bioassays, comparative LC-MS/MS secretome analysis, and activity-guided fractionation point to a blend of T2SS-secreted lytic enzymes (including chitinases, chitosanases, glucanases, and proteases) that causes mushroom degradation. In light of our results, we propose an updated model of soft rot disease in which degradation of the compact mushroom cell wall by these T2SS-secreted proteins likely facilitates access of jagaricin to the membrane (Fig. 4). In this way, *J. agaricidamnosum* joins the ranks of mushroom-, fruit-, and vegetable-infecting soft rot pathogens that follow a common strategy to evoke symptoms, i.e., secretion of hydrolytic enzymes by a T2SS (5, 49, 61).

Moreover, we show that the T3SS contributes significantly to mushroom degradation and present the first example of a T3SS involved in mushroom soft rot disease. We used bioinformatic analysis to uncover six potential T3 effectors with five of them resembling known effectors of phytopathogenic bacteria. However, the molecular details of how the T3SS-secreted proteins of *J. agaricidamnosum* drive disease progression remain to be deciphered. Since T3 effector proteins have long been recognized as important virulence factors of various plant-pathogenic bacteria (51, 63, 64), our discovery reveals a common principle of the pathobiology of mushroom- and plant-pathogenic bacteria. Remarkably, while secretion systems of plant-pathogenic Gram-negative bacteria are quite well studied, the role of secretion systems in mycopathogenic bacteria has remained underexplored. We addressed this knowledge gap and unveiled the previously elusive roles of *J. agaricidamnosum* secretion systems in mushroom soft rot disease. This finding opens up a new avenue of research into the development of secretion system inhibitors to disarm *J. agaricidamnosum*. We expect our work will eventually help mitigate the severe economic losses caused by mushroom soft rot disease.

## MATERIALS AND METHODS

### Bacterial strains and growth conditions

Strains used in this study are listed in Table S5 in the supplemental material. *Janthinobacterium agaricidamnosum* DSM 9628 was procured from the German Collection of Microorganisms (DSMZ). Routine cultivation was performed in modified nutrient media (DSMZ Medium 605; 1 g L^−1^ beef extract, 2 g L^−1^ yeast extract, 5 g L^−1^ peptone, and 15 g L^−1^ agar, pH 6.5) and CYM (2 g L^−1^ yeast extract, 2 g L^−1^ polypeptone, 20 g L^−1^ glucose, 15 g L^−1^ agar, pH 6.5). Solid and liquid cultures (with orbital shaking in baffled flasks) were incubated at room temperature and 26°C, respectively. Secretion system-deficient mutants were selected on modified nutrient media supplemented with 50 µg mL^−1^ kanamycin. *Escherichia coli* HST08 Stellar (Takara Bio, Saint-Germain-en-Laye, France) and *E. coli* ER2925 (New England Biolabs GmbH, Frankfurt am Main, Germany) were used to construct plasmids for gene disruption. *E. coli* strains were cultured at 37°C in lysogeny broth (LB; 10 g L^−1^ tryptone, 5 g L^−1^ yeast extract, 5 g L^−1^ NaCl, and 1 g L^−1^ glucose), supplemented with 5 µg mL^−1^ kanamycin for selection, whenever appropriate.

### Construction of plasmids for T2SS and T3SS gene disruption

Oligonucleotide primers and plasmids are listed in Tables S6 and S7, respectively. The mutation strategy was based on target gene disruption via homologous recombination inspired by previous studies (25, 52). Genomic DNA of *J. agaricidamnosum* was used as template to amplify a portion of the region encoding the targeted secretion system. In order to construct the vector pKO*gspE,* homologous regions (1,000 bp) downstream (hr1) and upstream (hr2) of the target gene *gspE* were amplified by PCR using the primer pair *gspE*_hr1_fw/*gspE*_hr1_rv and *gspE*_hr2_fw/*gspE*_hr2_rv, respectively, with 15bp extensions complementary to the linearized vector and 8 bp extensions complementary to the kanamycin-resistance gene *kan*^r^. Additionally, *kan*^r^ was amplified from the commercially available vector pK19 (65) with the primer pair *gspE*_*kan*^r^_fw/*gspE*_ *kan*^r^_rv that incorporates 8 bp extensions, homologous to hr1 and hr2 of the target region. All amplifications were carried out using Phusion High-Fidelity DNA Polymerase. The three resulting PCR fragments (hr1, hr2, and *kan*^r^) were assembled with *Sma*I-linearized pGL42a by using the In-Fusion HD Cloning Kit (Takara Bio), yielding the plasmid pKO*gspE*. The reaction mixture was subsequently used to transform chemically competent *E. coli* HST 08 Stellar cells. The plasmid was verified by restriction digest and Sanger sequencing of the cloned region using the primers M13_fw and M13_rv. Finally, the methylase-deficient *E. coli* strain ER2925 was transformed with pKO*gspE* by electroporation (2 mm cuvette, 2,500 V, 25 µF, and 200 Ω). The unmethylated plasmid pKO*gspE* was purified from overnight cultures using the Monarch Plasmid Miniprep Kit (New England Biolabs) and verified by restriction digest. The vector pKO*sctC* was analogously constructed. Briefly, homologous regions (1,000 bp) upstream and downstream of the target gene *sctC* were amplified by PCR using the primer pairs *sctC*_hr1_fw/*sctC*_hr1_rv and *sctC*_hr2_fw/*sctC*_hr2_rv, respectively. The resistance gene *kan*^r^ was amplified with the primers *sctC*_*kan*^r^_fw and *sctC*_*kan*^r^_rv. Assembly and cloning were performed as described above.

### Transformation of *J. agaricidamnosum*

Twenty milliliters of modified nutrient medium was inoculated with 1 mL of bacterial overnight preculture to give an optical density at 600 nm (OD_600_) of 0.1. The culture was incubated at 23°C with orbital shaking until an OD_600_ of 0.4 was reached. All subsequent steps were performed on ice. Cells were harvested by centrifuging at 4,000 × *g* for 5 minutes at 4°C. The pellet was washed twice with 300 mM sucrose and dissolved in 0.5 mL ice-cold sterile water. An aliquot of 60 µL competent cells was mixed with 20–200 ng of unmethylated pKO*gspE* or pKO*sctC* and electroporation was performed (2 mm cuvette, 2,500 V, 25 µF, and 200 Ω). Cells were incubated in CYM at 23°C with orbital shaking for 3 hours to recover, then spread onto modified nutrient agar plates supplemented with 50 µg mL^−1^ kanamycin. Colonies were screened for mutations by PCR of the relevant genomic regions and compared to the equivalent region amplified from the wild type by agarose gel electrophoresis.

### Button mushroom infection assay

*J. agaricidamnosum*, *J. agaricidamnosum* Δ*gspE* (deficient in T2SS), and *J. agaricidamnosum* Δ*sctC* (deficient in T3SS) were grown overnight in 20 mL modified nutrient medium in baffled flasks at 26°C with orbital shaking. Prior to inoculation, button mushrooms were cut into 0.5 cm thick slices, weighed, and placed in sterile petri dishes. To prevent the slices from drying out, a wet sterilized tissue was placed in the center of the plates. Inocula of 2.7 × 10^7^ cells were spotted onto two different areas of each trama. Slices inoculated with medium served as negative (untreated) controls. Plates were sealed and mushroom slices were incubated for 6 days at room temperature, then re-weighed. The experiments were done in quadruplicate and each experiment consisted of six biological replicates with four mushroom slices each. The disease symptoms were quantified by measuring the degradation of mushroom slices; specifically, the differences between the weights of mushroom tissue at the beginning versus at the end of the experiment were statistically analyzed using two-way analysis of variance (ANOVA) followed by Bonferroni’s mean comparison test as implemented in the GraphPad Prism 5 software. Raw data are listed in Tables S8 and S9.

### Preparation of colloidal chitin

One gram of practical grade shrimp shell chitin (Sigma C9213) was added to 40 mL of 37% HCl at room temperature and stirred for 2 hours. This solution was poured into 1 L of cold distilled water (dH_2_O) and washed several times until the pH was neutral. After filtration (Miracloth), the chitin pellet was lyophilized, autoclaved, and stored at 4°C until use.

### *In vivo* activity assay

In order to determine if *J. agaricidamnosum* and *J. agaricidamnosum* Δ*gspE* have proteolytic or chitosan-/chitin-degrading activity, the strains were individually grown overnight in 2 mL modified nutrient medium, then centrifuged for 2 minutes at 4,000 × *g*. The cultures were washed and diluted in fresh nutrient medium. A loopful of *J. agaricidamnosum* or *J. agaricidamnosum* Δ*gspE* was streaked onto agar plates where half the area consisted of nutrient agar while the other was filled with either milk agar (milk powder 10 g L^−1^ and 1% agarose) or chitin agar [10 g L^−1^ acid-swollen chitin, 3 g L^−1^ (NH_4_)_2_SO_4_, 2 g L^−1^ KH_2_PO_4_, 0.3 g L^−1^ MgSO_4_ × 7 H_2_O, Calcofluor White MR2 (Sigma-Aldrich Chemie GmbH, Schnelldorf, Germany) 1% (vol/vol), pH 6.5]. The plates were incubated at room temperature for 3 days.

### Bioinformatic analyses

Geneious Prime 2022 was used to visualize the annotated *Janthinobacterium agaricidamnosum* genome, which was manually browsed for secretion system gene clusters. Obscure gene products encoded adjacent and within the secretion system gene clusters were analyzed for their similarity to characterized proteins using both HHpred (30) and NCBI BLAST (29) and re-annotated. Putative effector proteins were predicted using the EFFECTIVE T3 prediction tool (35). Potential exoproteins encoded in the *J. agaricidamnosum* genome were manually screened using a set of computational methods for prediction. Secretion signal peptides (SPs) and twin-arginine translocation (tat) signal peptides were predicted with SignalP 6.0 (66). Alternative secretion was predicted with SecretomeP 2.0 (67). Putative exoproteins were characterized using InterPro (68) and HHpred (30).

### Bioactivity-guided protein fractionation

In order to find proteins that degrade mushroom tissue, *J. agaricidamnosum* and *J. agaricidamnosum* Δ*gspE* were cultivated in mushroom medium (0.5 g L^−1^ lyophilized, ground brown button mushrooms, pH 6.5) at 26°C with orbital shaking overnight. The cultures were centrifuged at 6,000 × *g* for 10 minutes and the supernatants were filtered through a 0.22 µm filter. Ground (NH_4_)_2_SO_4_ was added stepwise to the supernatant (20%–80%). The precipitated proteins were collected by centrifugation at 12,000 × *g* for 10 minutes and dissolved in 200 µL water. After dialysis, 10 µL of the resolubilized protein fractions were spotted on two different areas of button mushroom slices and incubated for 1 day at room temperature. Additionally, the fractions were tested in a calcofluor chitinase assay. Heat-treated fractions were used as negative controls.

All protein fractions were further analyzed by SDS-PAGE and tryptic digestion as previously described (69). The proteins were digested with 3 µL Trypsin/Lys-C (Promega GmbH, Walldorf, Germany) solution (25 ng µL^−1^ in 10 mM NH_4_HCO_3_) and incubated at 4°C for 45 minutes. Excess solution was removed and 3 µL 10 mM NH_4_HCO_3_ were added. The gel particles were incubated at 37°C for 18 hours. The peptides were extracted stepwise with 50 µL of 1/49/50 trifluoroacetic acid (TFA)/H_2_O/ACN (vol/vol/vol), 50 µL of 1/29/70 TFA/H_2_O/ACN (vol/vol/vol), and 50 µL of 1/9/90 TFA/H_2_O/ACN (vol/vol/vol). After each step, the gels were sonicated in the water bath for 15 minutes and the supernatants were collected and pooled. The proteins were dried in a SpeedVac and stored at −80°C.

For MALDI-TOF-MS measurements, samples were solubilized in 4 µL of 0.05% TFA in 50% (vol/vol) ACN, then mixed with 52 mM α-cyano-4-hydroxycinnamate (HCCA) at a ratio of 1/1 and 2 µL of this mixture was spotted onto a MALDI plate. Samples were analyzed using a Bruker Daltonics UltrafleXtreme MALDI-TOF mass spectrometer. Spectra were obtained in reflector positive mode and the instrument was calibrated to a commercially available standard (Protein Calibration Standard II, Bruker Daltonics GmbH & Co. KG, Bremen, Germany) prior to each measurement. Data analysis was performed with flexAnalysis 3.3 (Bruker). Mass spectra were manually searched against *in silico* cleaved putative exoproteins of *J. agaricidamnosum* DSM 9628 using the PeptideMass tool on the ExPASy Server (70). Up to two missed cleavages were allowed for the tryptic digestion. Modifications were defined as methionine oxidation and cysteine carbamidomethylation.

### Purification of secreted proteins from *J. agaricidamnosum* and *J. agaricidamnosum* Δ*gspE* for LC-MS/MS

In order to identify secreted proteins, 100 mL mushroom medium (0.5 g L^−1^ lyophilized, ground brown button mushrooms, pH 6.5) was inoculated with 0.5 mL overnight cultures of *J. agaricidamnosum* or *J. agaricidamnosum* Δ*gspE* and incubated overnight. Cultures were pelleted by centrifugation at 6,000 × *g* for 10 minutes at 4°C and the supernatants were filtered through a 0.22 µm filter. Subsequently, TFA was added to a final concentration of 0.1% (vol/vol). Proteins were purified using a Supelco Supelclean LC-4 SPE Cartridge (Supelco, Schnelldorf, Germany) that had been prepared by washing with 4 mL ACN and reconstituting with 4 mL 0.1% (vol/vol) TFA. The supernatants were aspirated through the column, then the column was washed with 4 mL of 5% (vol/vol) MeOH in 0.1% (vol/vol) TFA. The samples were eluted with 4 mL 0.1% (vol/vol) TFA in 80% (vol/vol) ACN, then the eluates were evaporated in a vacuum centrifuge to near-dryness and stored overnight at −80°C. Afterward, the samples were resolubilized in 100 µL denaturation buffer (50 mM triethylammonium bicarbonate (TEAB) in 50% (vol/vol) 2,2,2-trifluoroethanol (TFE) and subjected to ultrasonication in a water bath for 15 minutes at room temperature. The samples were reduced and alkylated simultaneously at 70°C with orbital shaking in the dark for 30 minutes after adding 2 µL each of 500 mM tris(2-carboxyethyl)phosphine and 625 mM 2-chloroacetamide. Subsequently, proteins were precipitated as previously described (71). The resulting protein precipitate was resolubilized in 100 µL 100 mM TEAB in 5% (vol/vol) TFE and sonicated for 15 minutes. The total protein concentration was determined by the Merck Millipore Direct Detect System. The protein samples were diluted with 100 mM TEAB in 5% (vol/vol) TFE to give a final protein concentration of approximately 1 µg µL^−1^, then treated with 2 µg Trypsin/Lys-C protease mix per 50 µg protein sample for 18 hours at 37°C. The reaction mixture was evaporated in a vacuum centrifuge to dryness and resolubilized in 30 µL of 0.05% (vol/vol) TFA in 2% (vol/vol) ACN, followed by sonication in the water bath for 15 minutes. Peptides were transferred to a 10 kDa molecular weight cut off filter (VWR, Darmstadt, Germany) and centrifuged at 18,000 × *g* for 15 minutes at 4°C. The experiments were performed with three biological replicates and the filtrates were transferred to HPLC vials and stored at −20°C until LC-MS/MS analysis.

### LC-MS/MS secretome analysis

LC-MS/MS analysis was performed using an Ultimate 3000 Nano RSLC system connected to a Q Exactive HF mass spectrometer (both Thermo Fisher Scientific, Waltham, MA, USA). Peptide trapping for 5 minutes on an Acclaim Pep Map 100 Column (2 cm × 75 µm, 3 µm particle size) at 5 µL minutes^−1^ was followed by separation on an analytical Acclaim Pep Map RSLC Cano Column (50 cm × 75 µm, 2 µm). Eluent A was 0.1% (vol/vol) formic acid, eluent B was 0.1% (vol/vol) formic acid in 90% (vol/vol) ACN. Mobile phase gradient elution was performed as follows: 0–5 minutes at 4% B, 30 minutes at 7% B, 60 minutes at 10% B, 100 minutes at 15% B, 140 minutes at 25% B, 180 minutes at 45% B, 200 minutes at 65% B, 210–215 minutes at 96% B, and 215.1–240 minutes at 4% B.

Positively charged ions were generated at a spray voltage of 2.2 kV using a stainless-steel emitter attached to the Nanospray Flex Ion Source (Thermo Fisher Scientific). The quadrupole/orbitrap instrument was operated in Full MS/data-dependent MS2 Top 15 mode. Precursor ions were monitored at *m*/*z* 300–1500 at a resolution of 120,000 FWHM (full width at half maximum) using a maximum injection time (ITmax) of 140 ms and an AGC (automatic gain control) target of 3 × 10^6^. Precursor ions with a charge state of *z* = 2–5 were filtered at an isolation width of *m*/*z* 2.0 amu for further HCD fragmentation at 30% normalized collision energy (NCE). MS^2^ ions were scanned at 15,000 FWHM, ITmax of 140 ms and an AGC target of 2 × 10^5^. Dynamic exclusion of precursor ions was set to 30 seconds.

### Protein database search and data analysis

Tandem mass spectra were searched against the UniProt databases of *Janthinobacterium agaricidamnosum* DSM 9628 (https://www.uniprot.org/proteomes/UP000027604) and *Agaricus bisporus* (https://www.uniprot.org/proteomes/UP000629468) from 22/08/11 (YYMMDD) using Thermo Scientific Proteome Discoverer (PD) 2.4 and the algorithms of Mascot 2.8, Sequest HT, MS Amanda 2.0, and MS Fragger 3.4. Two missed cleavages were allowed for the tryptic digestion. The precursor mass tolerance was set to 10 ppm and the fragment mass tolerance was set to 0.02 Da. Modifications were defined as dynamic methionine (Met) oxidation and protein N-terminal Met-loss and/or acetylation as well as static cysteine carbamidomethylation. A strict false discovery rate (FDR) <1% (peptide and protein level) and search engine thresholds of >30 (Mascot), >4 (Sequest HT), >300 (MS Amanda), and >8 (MS Fragger) were required for positive protein hits. The Percolator node and a reverse decoy database was used for q-value validation of spectral matches. Only rank 1 proteins and peptides of the top scored proteins were counted. Label-free protein quantification was based on the Minora algorithm of PD2.4 using a signal-to-noise ratio of >5. The following requirements needed to be met to qualify as a significant change in protein abundance between *J. agaricidamnosum* and *J. agaricidamnosum* Δ*gspE*: a fold change greater than 2 or less than −2, a *P*-value from two-sided *t*-test divided by the absolute value of the log_4_ ratio [*P*-value/ABS(log_4_ ratio)] <0.05. Furthermore, the protein should be identified in at least two of three biological replicates of the sample group with the highest protein abundance.

## Data Availability

The mass spectrometry proteomics data have been deposited to the ProteomeXchange Consortium via the PRIDE (72) partner repository with the data set identifier PXD040852.

## SUPPLEMENTAL MATERIAL

Supporting Information mbio.00787-23-s0001.pdf Supplemental figures, tables, and procedures.

ASM does not own the copyrights to Supplemental Material that may be linked to, or accessed through, an article. The authors have granted ASM a non-exclusive, world-wide license to publish the Supplemental Material files. Please contact the corresponding author directly for reuse.
