# Supplementary material for: Bacterial secretion systems contribute to rapid tissue decay in button mushroom soft rot disease
Source: mBio. 2023 Jul 24;14(4):e00787-23. doi: 10.1128/mbio.00787-23 (PMC10470514; doi:10.1128/mbio.00787-23)
Supplement: Supporting Information — Supplemental figures, tables, and procedures. [file mbio.00787-23-s0001.pdf]

Supporting Information

Bacterial Secretion Systems Contribute to Rapid Tissue Decay in Button Mushroom Soft Rot Disease

Philipp Wein, Katharina Dornblut, Sebastian Herkersdorf, Thomas Krüger, Evelyn M. Molloy, Axel A. Brakhage, Dirk Hoffmeister, Christian Hertweck

This file includes:

Experimental Section

Figures S1 to S16

Tables S1 to S9

References

Supplementary Information

Experimental Section

Figures

Fig. S1 Genetic organization of type III secretion systems from *Janthinobacterium* species
Fig. S2 Evolutionary relationship between alanine-tryptophan-arginine triad (AWR) effectors
Fig. S3 Multiple sequence alignment of predicted AWR effector proteins
Fig. S4 Sequence alignment of conserved regions in AWR proteins
Fig. S5 Gene expression analyses of T2SS and T3SS related genes
Fig. S6 Verification of the mutant Δ*gspE*
Fig. S7 Verification of the mutant Δ*sctC*
Fig. S8 Growth kinetics of *J. agaricidamnosum* wild type, Δ*gspE* and Δ*sctC*
Fig. S9 Swarming assay of *J. agaricidamnosum* wild type and deletion mutants
Fig. S10 Bioactivity-guided protein fractionation
Fig. S11 SDS-PAGE analysis of protein fractions
Fig. S12 MALDI-TOF-MS and domain analysis of protein 1
Fig. S13 MALDI-TOF-MS and domain analysis of protein 2
Fig. S14 MALDI-TOF-MS analysis of protein 3
Fig. S15 MALDI-TOF-MS analysis of protein 4
Fig. S16 MALDI-TOF-MS and domain analysis of protein 5

Tables

Table S1. Secretion associated genes and proposed function of encoded proteins
Table S2. Potentially relevant T3 effector proteins encoded within the T3SS gene cluster
Table S3. Proteins identified by bioactivity-guided fractionation
Table S4. First ten results returned in a HHpred search of the RICIN domain-containing protein (CDG82784)
Table S5. Strains used in this study
Table S6. Oligonucleotide primers used in this study
Table S7. Plasmids used in this study
Table S8. Quantification of mushroom (*A. bisporus*) degradation caused by *J. agaricidamnosum* strains
Table S9. Statistical analysis of mushroom degradation caused by *J. agaricidamnosum* strains

References

Phylogenetic analysis

The accession numbers of sequences used for the alignment and tree construction are shown in Figures S2–S4.The protein sequence of the putative type III-secreted AWR effector from *J. agaricidamnosum* was aligned with those of homologs from plant pathogenic *R. solanacearum*, *Burkholderia* spp., and *Xanthomonas* strains using Clustal Omega 1.2.2 as implemented in the Geneious Prime 2022 Software (1). The phylogenetic tree was constructed in MEGA7 (2) using the Neighbor-Joining method (3) with 10,000 bootstrap repetitions (4). Evolutionary distances were computed using the Poisson correction method (5). Positions containing gaps and missing data were eliminated.

Gene expression analyses

Oligonucleotide primers are listed in Table S6. RNA isolation was achieved by resuspending the cell pellet obtained from 2 mL overnight culture in 1 mL TRIsure^{TM} RNA isolation reagent (Bioline). Cell disruption was carried out in lysis tubes (Biospec Products) using a SpeedMill PLUS homogenizer (analytikjena), before following the procedure outlined in the TRIsure manual. Remaining DNA was removed with Turbo-DNAse (Ambion) and the absence of DNA in the isolated RNA was confirmed using PCR. The Omniscript Reverse Transcription (RT) Kit (QIAGEN) was used for the reverse transcription reaction at 37 °C for 1 h. Fragments of T2SS-related genes (*gspD*, *gspE*) and T3SS-associated genes (*sctC*, *sctT*) were amplified using the KAPA2G Robust DNA Polymerase (Kapa Biosystems) and the respective primers. The housekeeping gene *rpoB* was used as positive control.

Growth kinetics of *J. agaricidamnosum* strains

Overnight cultures of *J. agaricidamnosum* and the deletion mutants *J. agaricidamnosum* Δ*gspE* and *J. agaricidamnosum* Δ*sctC* were individually added to 30 mL volumes of modified nutrient medium (DSMZ 605 medium) or CYM (in 100 mL baffled flasks) to give an optical density at 600 nm (OD_{600}) of 0.1. The cultures were incubated at 26 °C with orbital shaking, and the OD_{600} was measured periodically for 30 h. The experiments were performed in triplicates.

Source of mushroom fruiting bodies

Healthy organic fruiting bodies of the brown button mushroom *Agaricus bisporus* were purchased from local supermarkets and were of various geographical origins.

Swarming assay

Overnight cultures (2 mL) of *J. agaricidamnosum* and the deletion mutants *J. agaricidamnosum* Δ*gspE* and *J. agaricidamnosum* Δ*sctC* were centrifuged, then the cell pellets were resuspended in modified nutrient medium to give an OD_{600} of 1. An aliquot (10 µL) of each culture was spotted centrally of LB soft agar (0.6 % agar). Plates were sealed with parafilm and incubated at room temperature for 48 h. Each experiment was performed with three biological replicates and documented photographically.

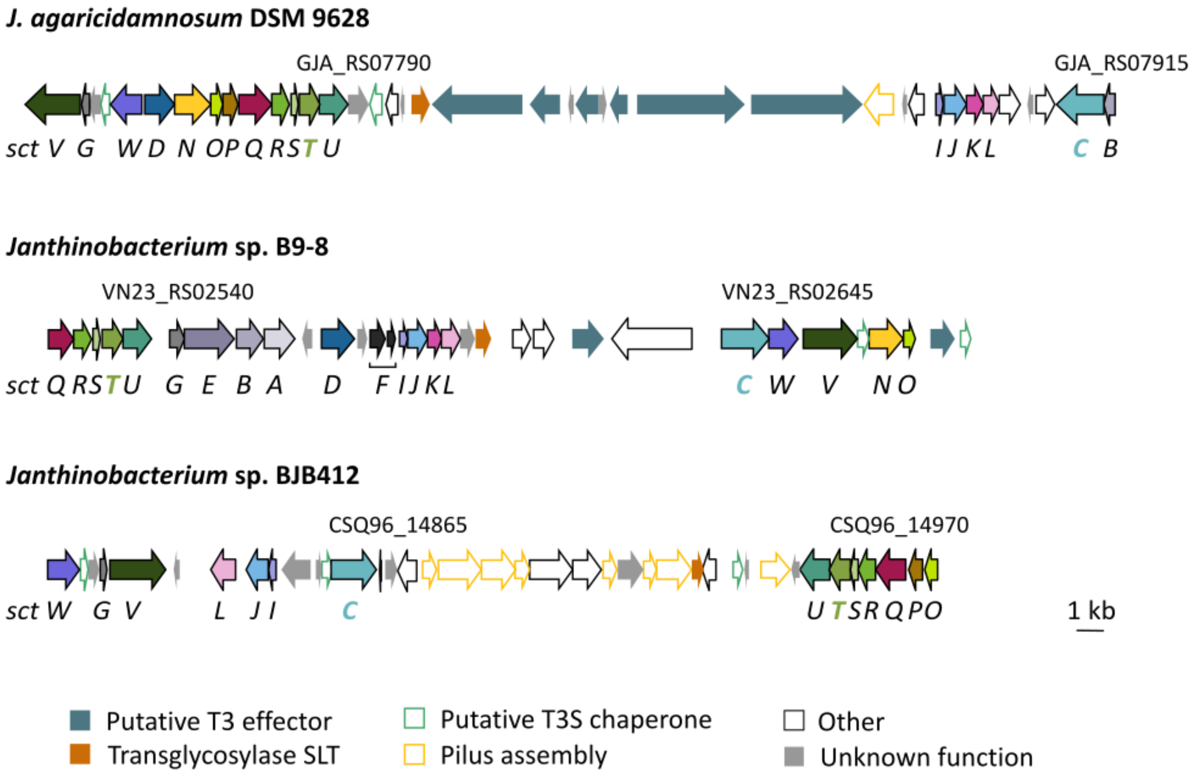

Fig. S1 Genetic organization of type III secretion systems (T3SSs) from *Janthinobacterium* species. In order to reveal the distribution of T3SS gene clusters within the genus *Janthinobacterium*, the proteins encoded by *sctC* (GJA_RS07790) and *sctT* (GJA_RS07915) from *J. agaricidamnosum* DSM 9628 were used in a protein BLAST search against the *Janthinobacterium* (taxid:29580) database. T3SS gene clusters were found in the genome of *Janthinobacterium* sp. B9-8 and *Janthinobacterium* sp. BJB412. In some cases, genes were re-annotated/annotated with HHpred (6) and NCBI BLAST (7). Putative T3 effectors were predicted using the EFFECTIVE T3 software (8). Genes of T3SS core components are represented by arrows with letter designations. The predicted functions of other gene products are indicated in the key. Gene accession numbers are given for *sctC* and *sctT* genes of each cluster.

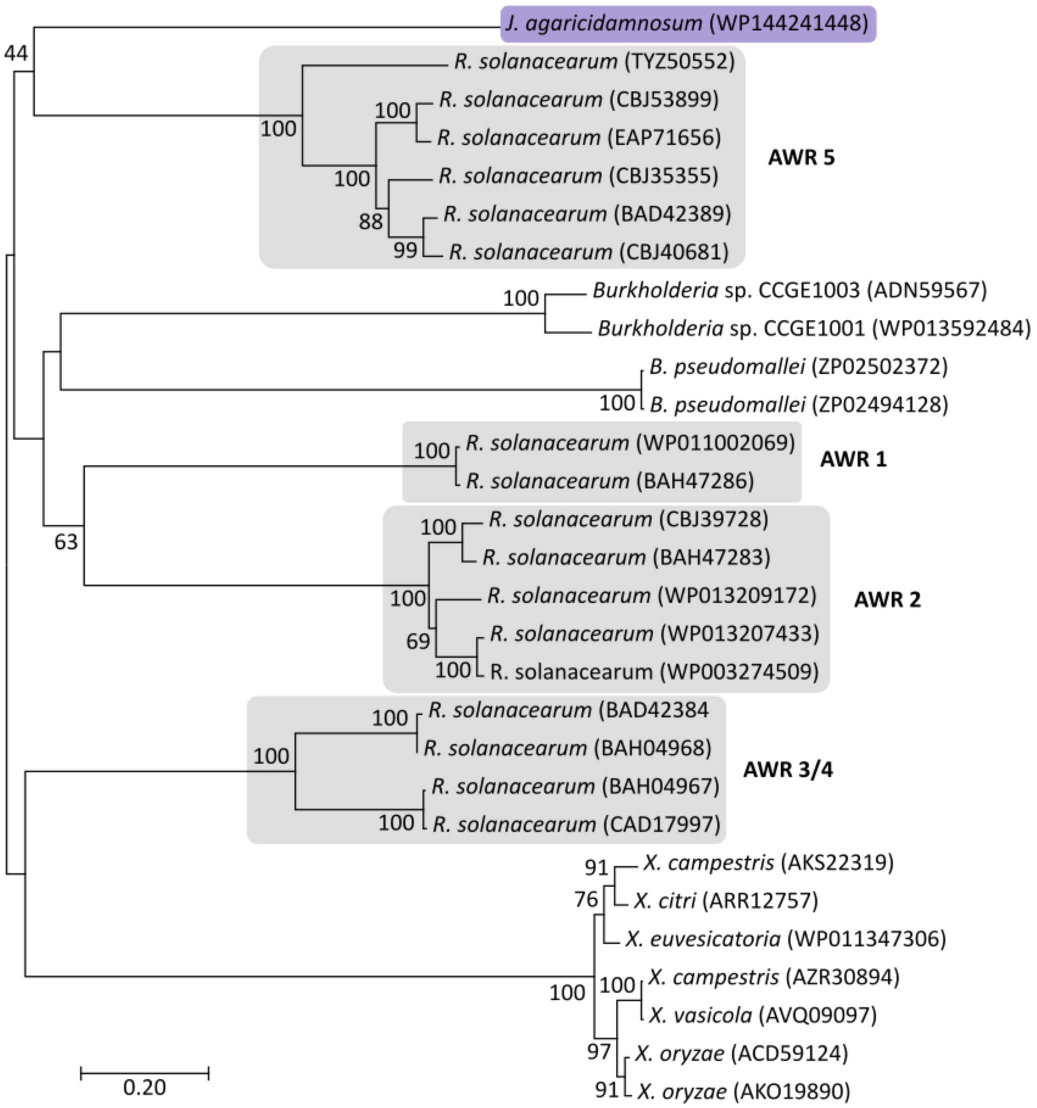

Fig. S2 Evolutionary relationship between alanine-tryptophan-arginine triad (AWR) effectors. The result of the phylogenetic analysis displays the AWR effector protein from the button mushroom pathogen *J. agaricidamnosum* (purple) and previously described AWR effectors from *Burkholderia* spp., and plant pathogenic *R. solanacearum* and *Xanthomonas* spp. (9). The putative AWR effector from *J. agaricidamnosum* belongs neither to the AWR family (AWR 1–5) of *R. solanacearum* nor to the other known AWR members. The analysis was conducted in MEGA7 (2) using the Neighbor-Joining algorithm (3). NCBI accession numbers are displayed in brackets.

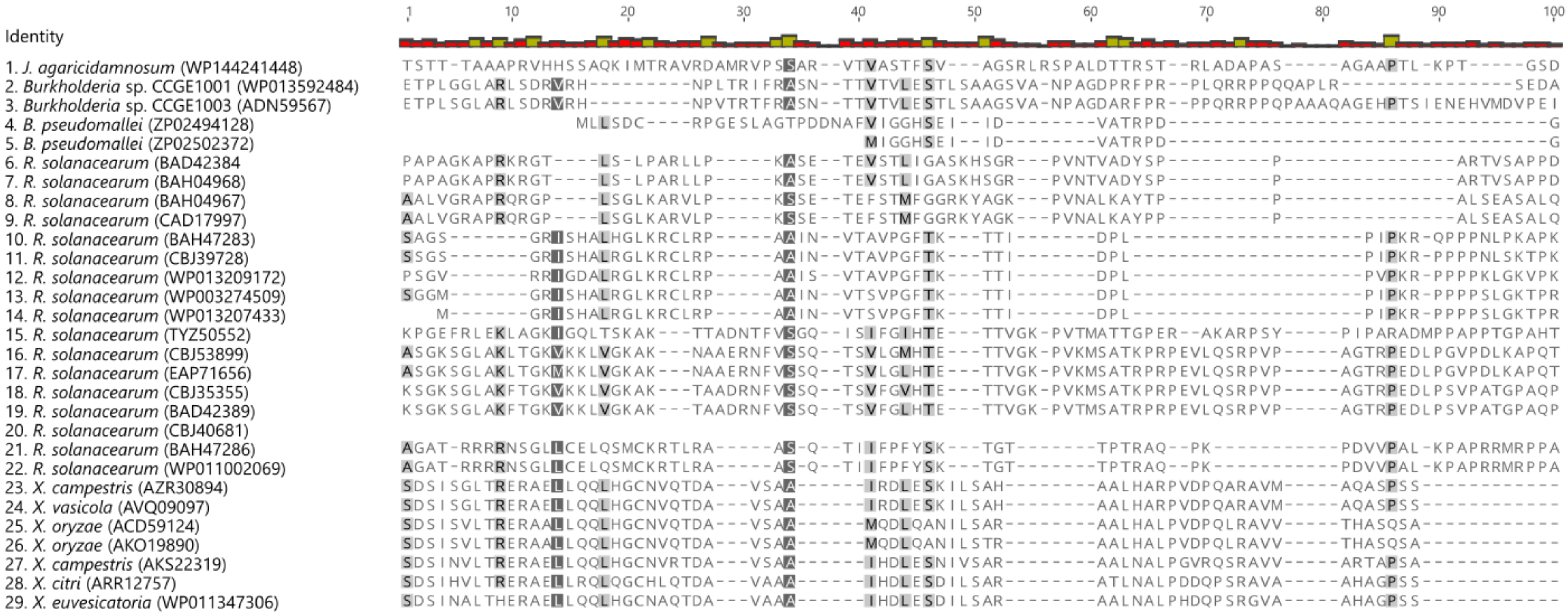

Fig. S3 Multiple sequence alignment of predicted AWR effector proteins. The alignment depicted is an updated version of the alignment from Sole *et al*. (9) and comprises AWR proteins from the button mushroom pathogen *J. agaricidamnosum*, plant pathogenic *R. solanacearum* strains, plant pathogenic *Xanthomonas* spp., and *Burkholderia* spp. Bar charts above the alignment display the overall sequence identity from low (red) to high (green). NCBI accession numbers are shown in brackets.

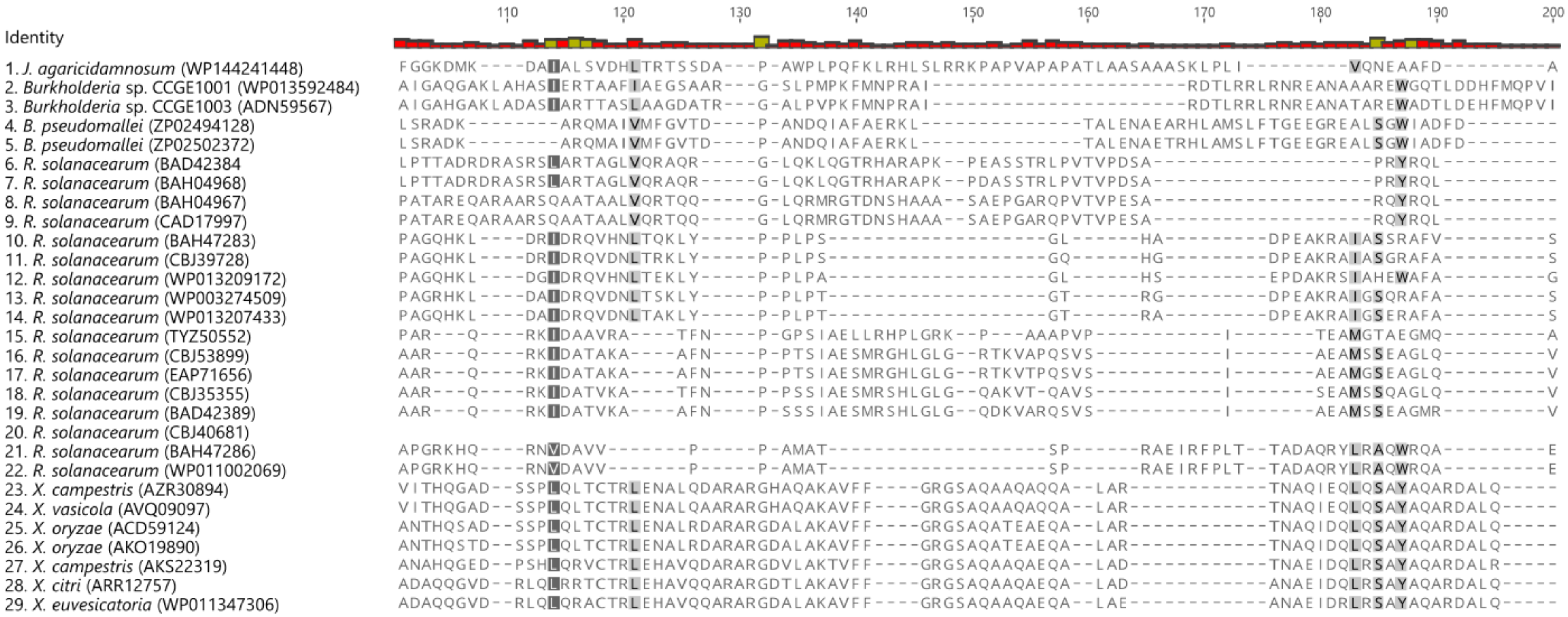

Fig. S3 (continued)

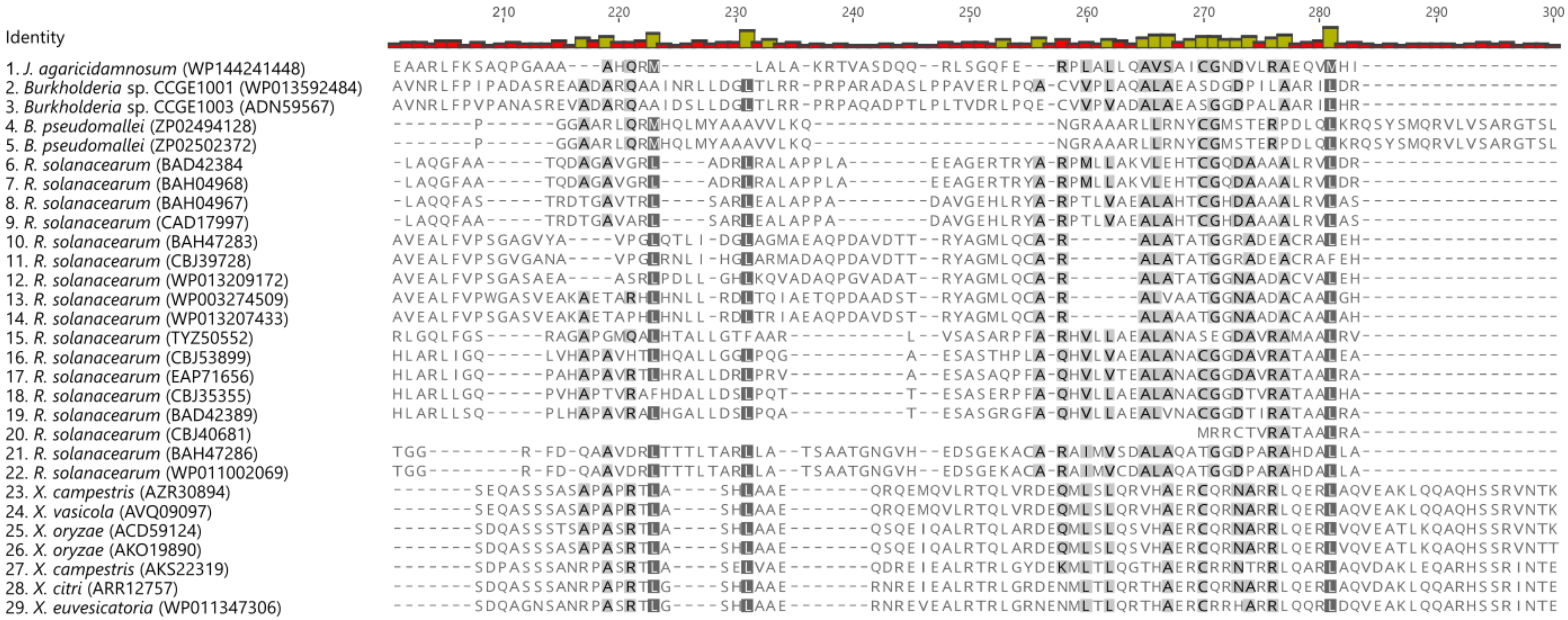

Fig. S3 (continued)

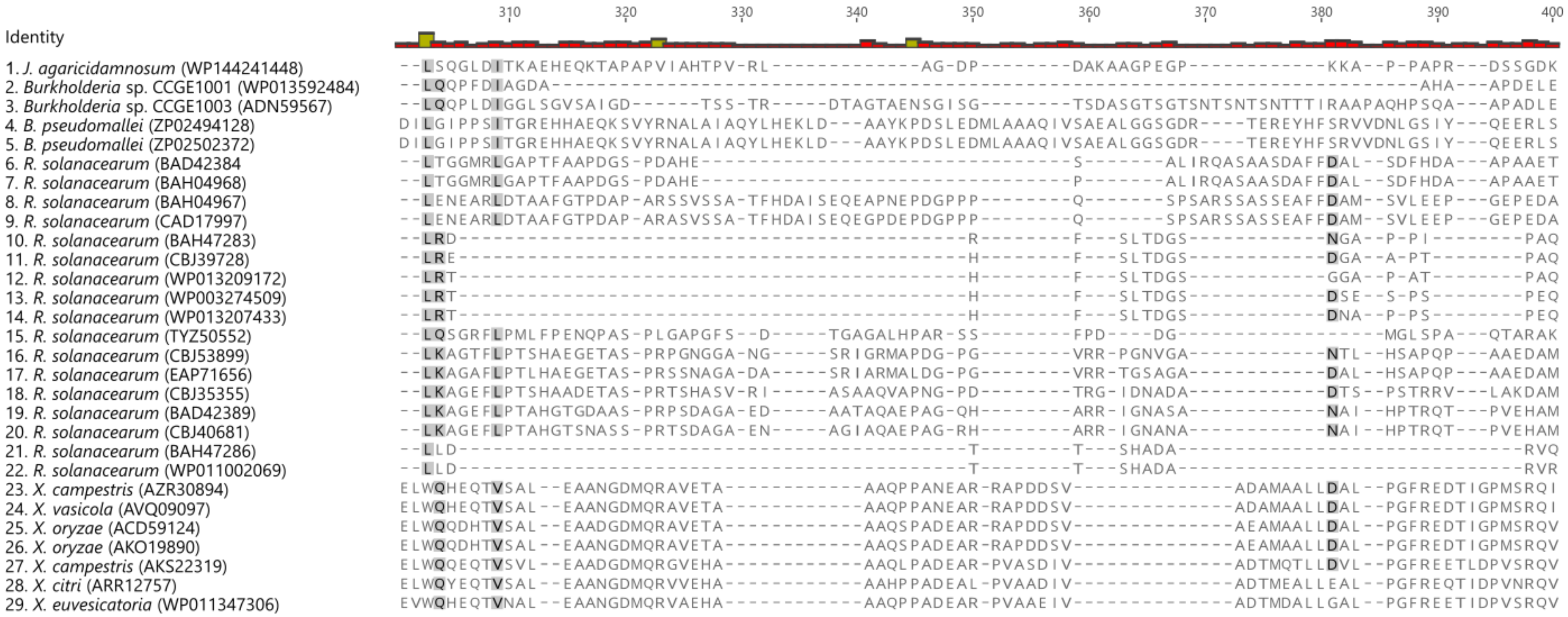

Fig. S3 (continued)

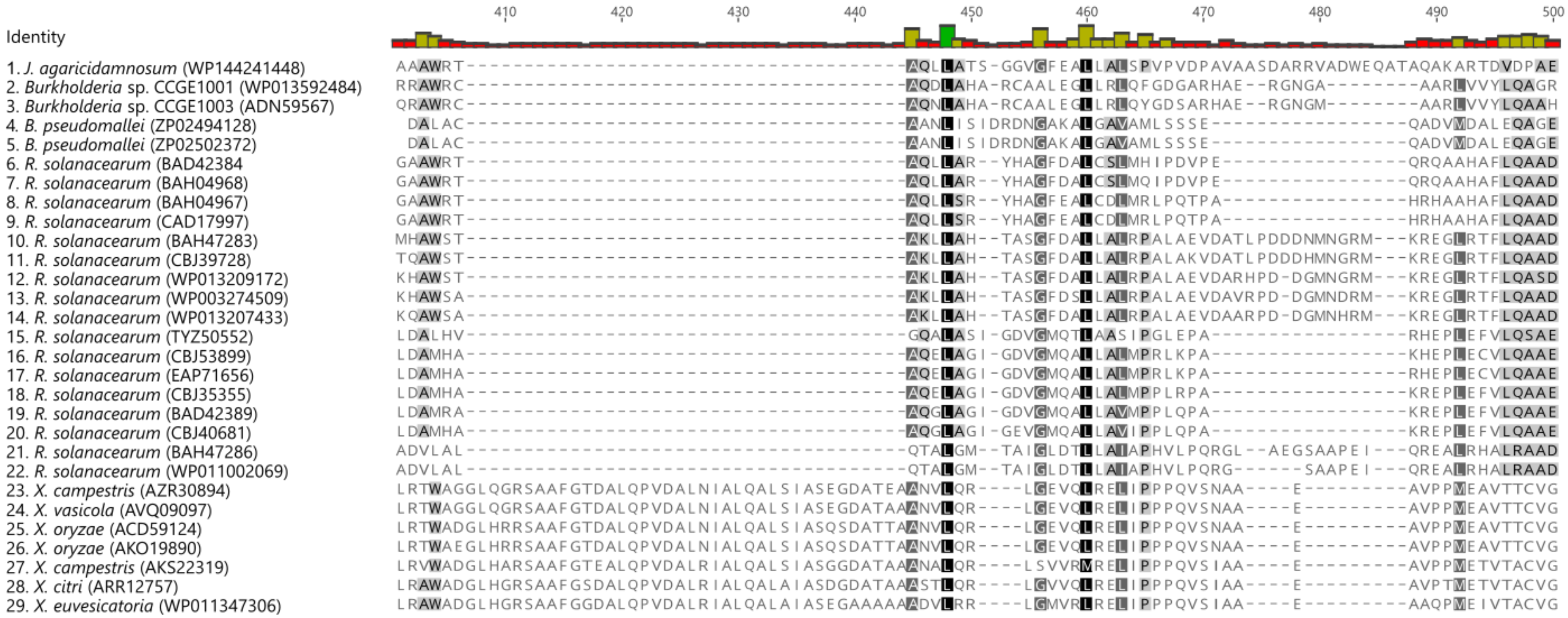

Fig. S3 (continued)

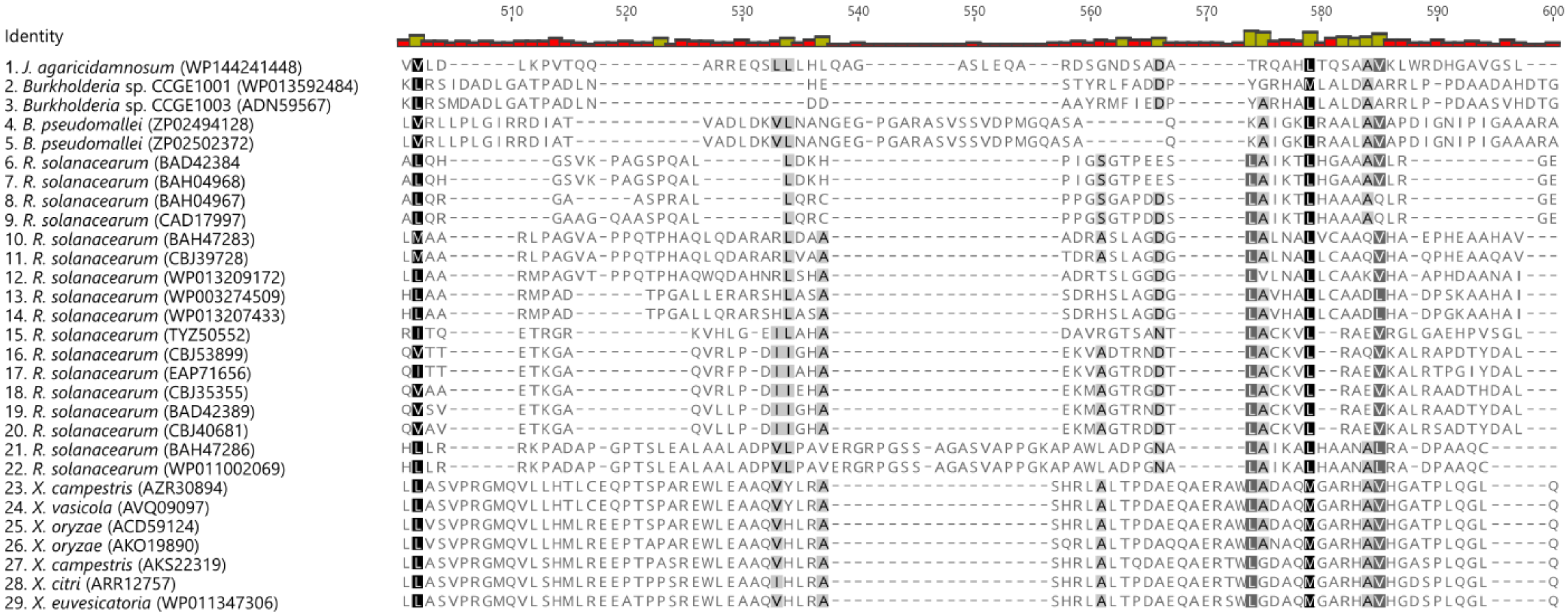

Fig. S3 (continued)

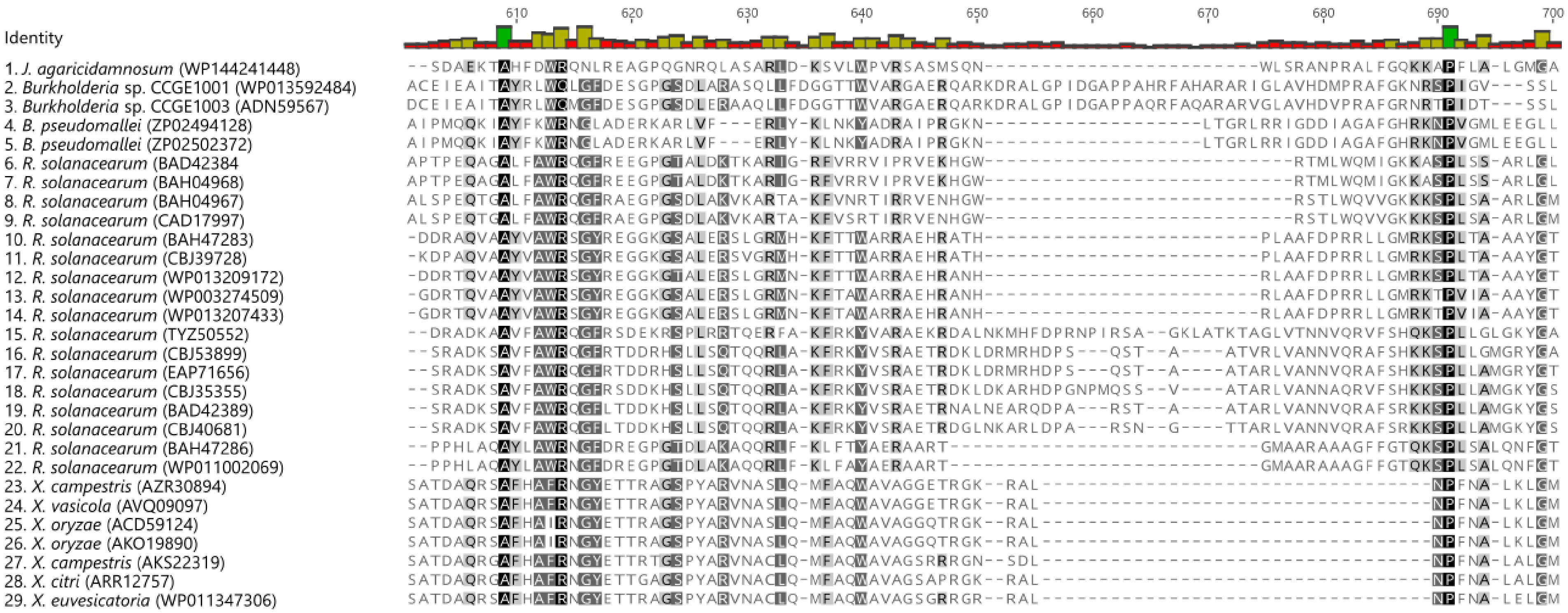

Fig. S3 (continued)

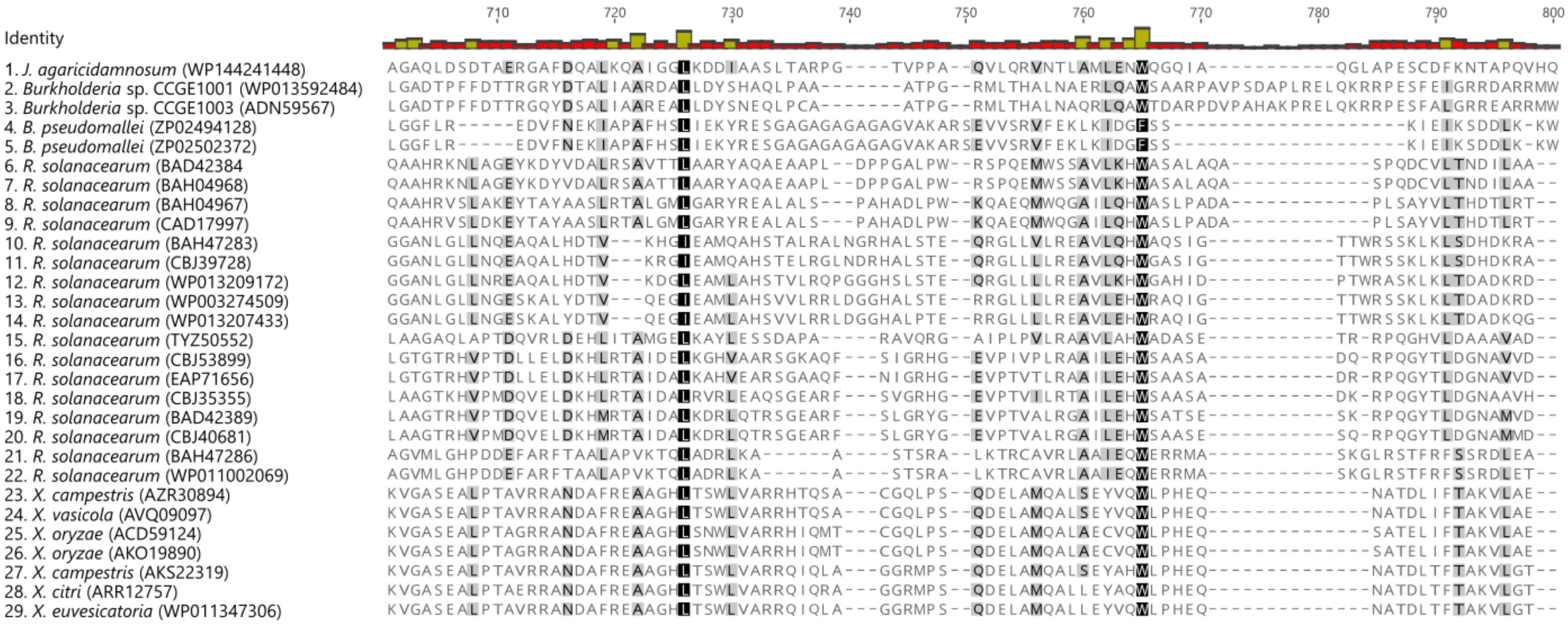

Fig. S3 (continued)

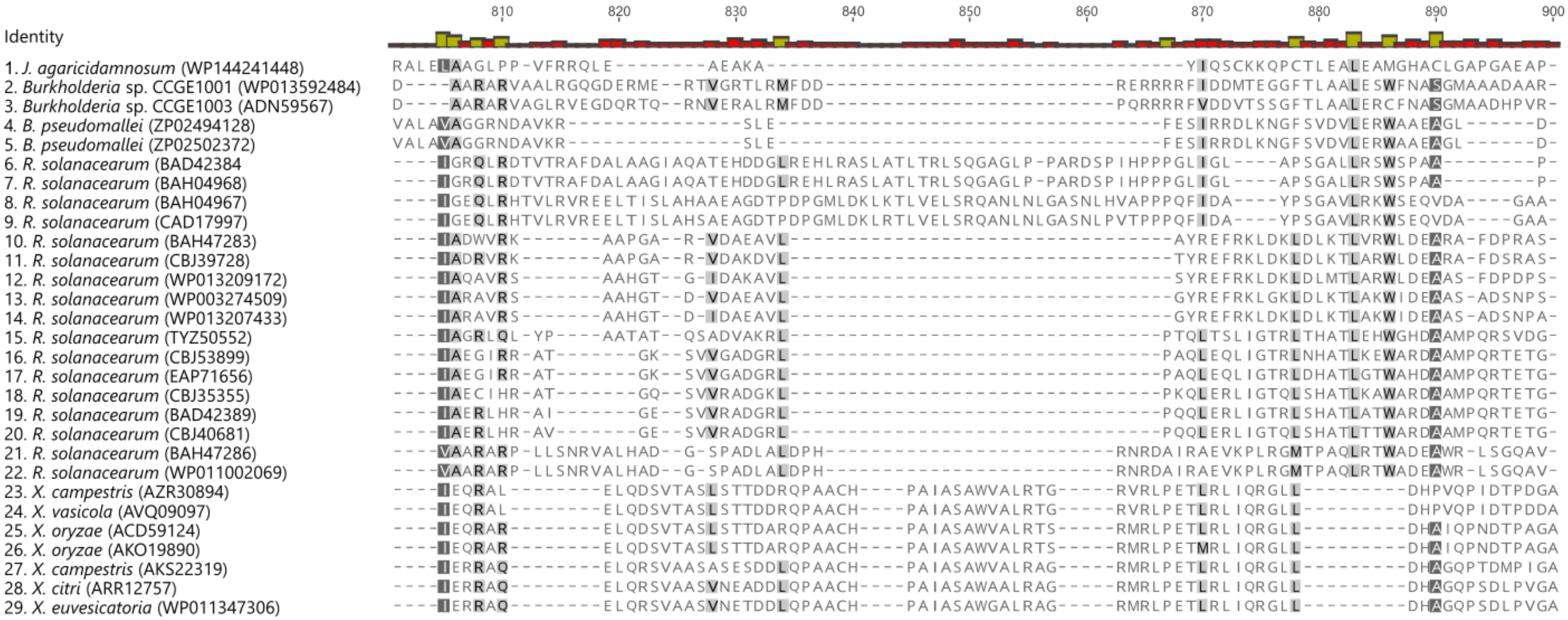

Fig. S3 (continued)

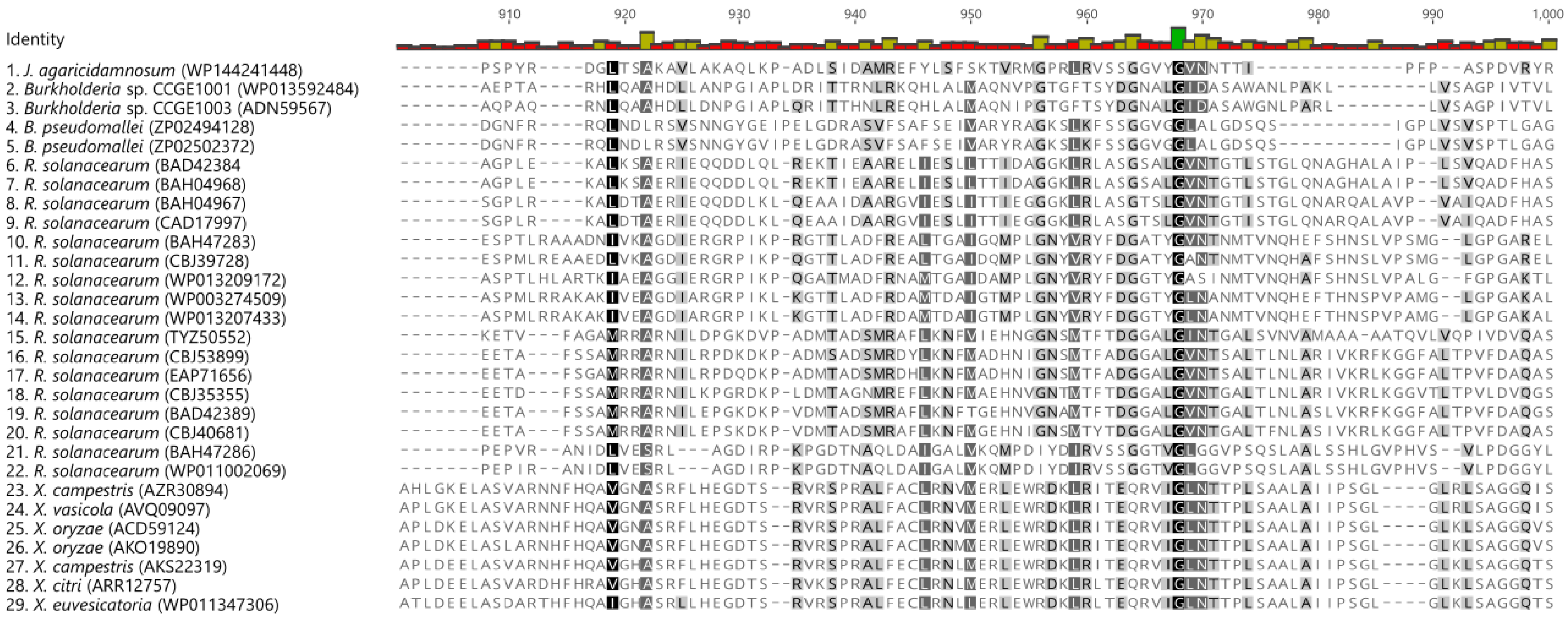

Fig. S3 (continued)

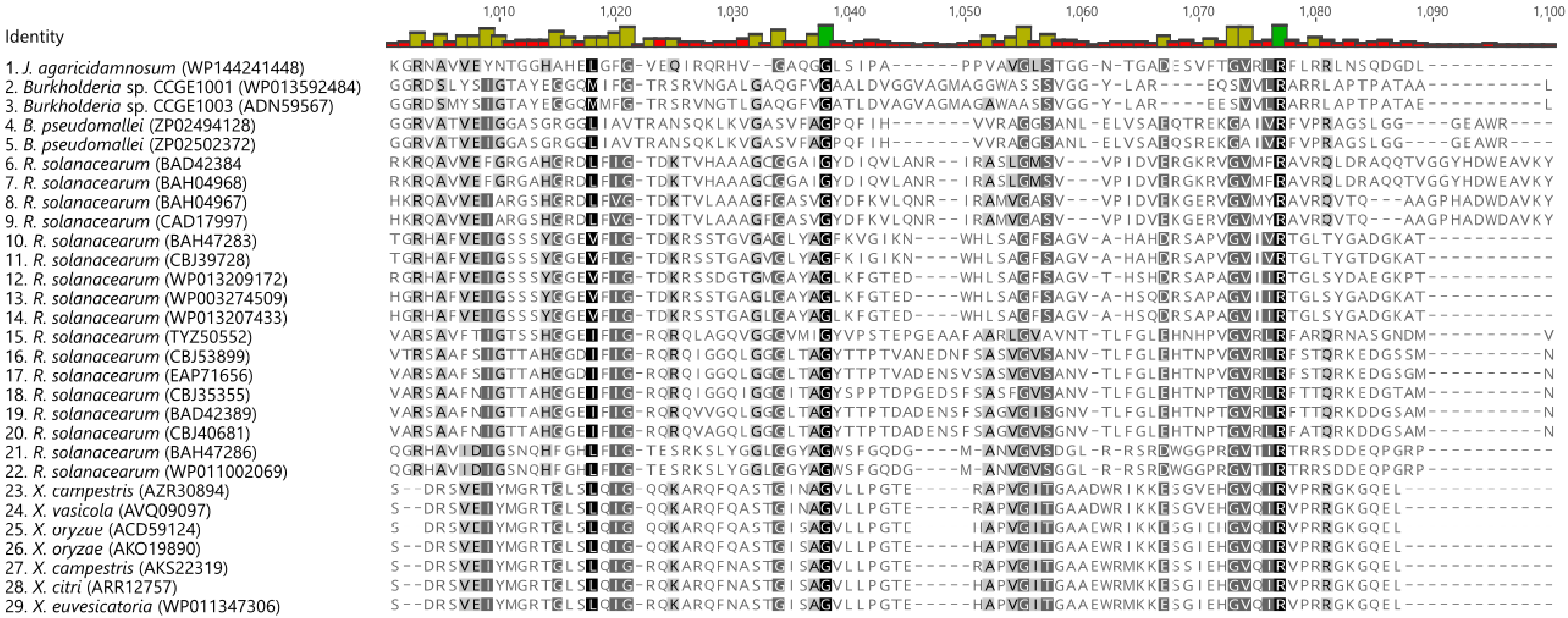

Fig. S3 (continued)

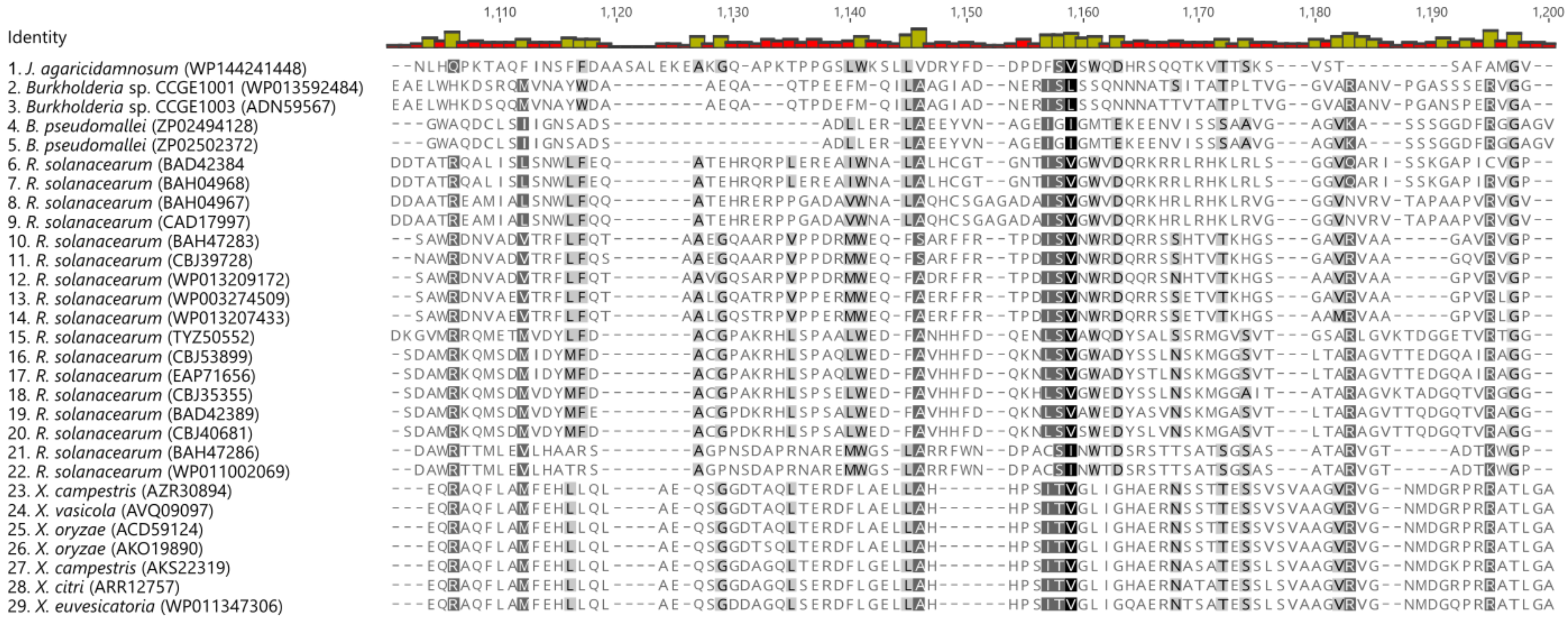

Fig. S3 (continued)

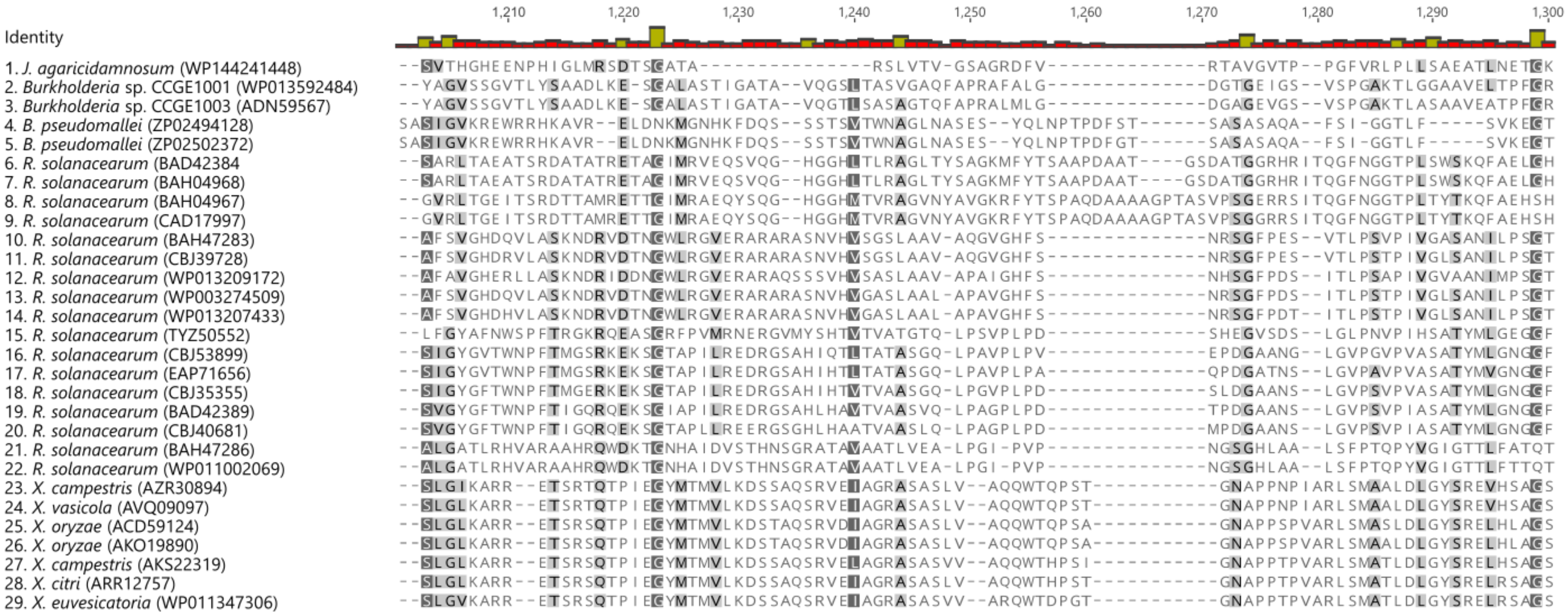

Fig. S3 (continued)

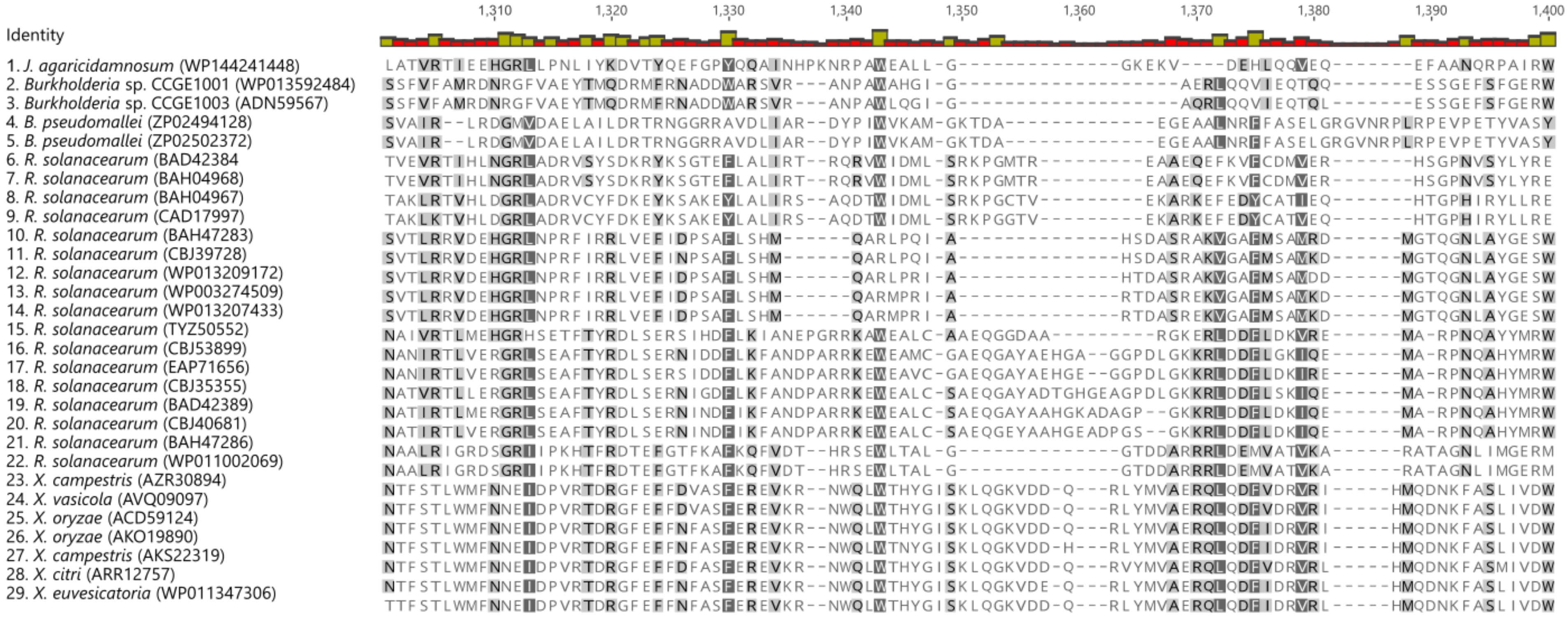

Fig. S3 (continued)

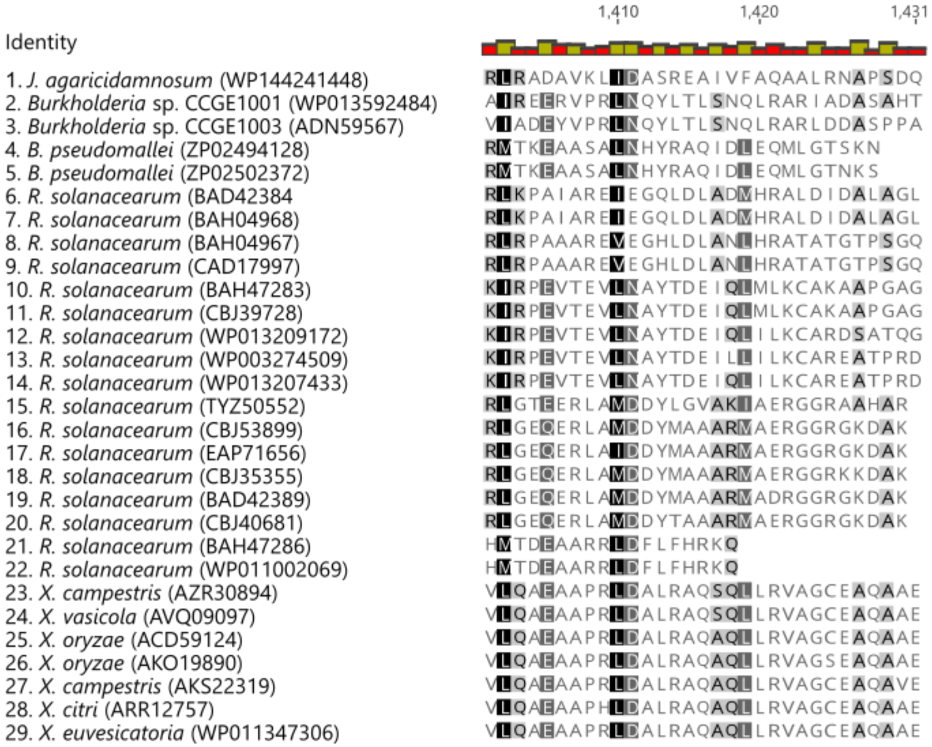

Fig. S3 (continued)

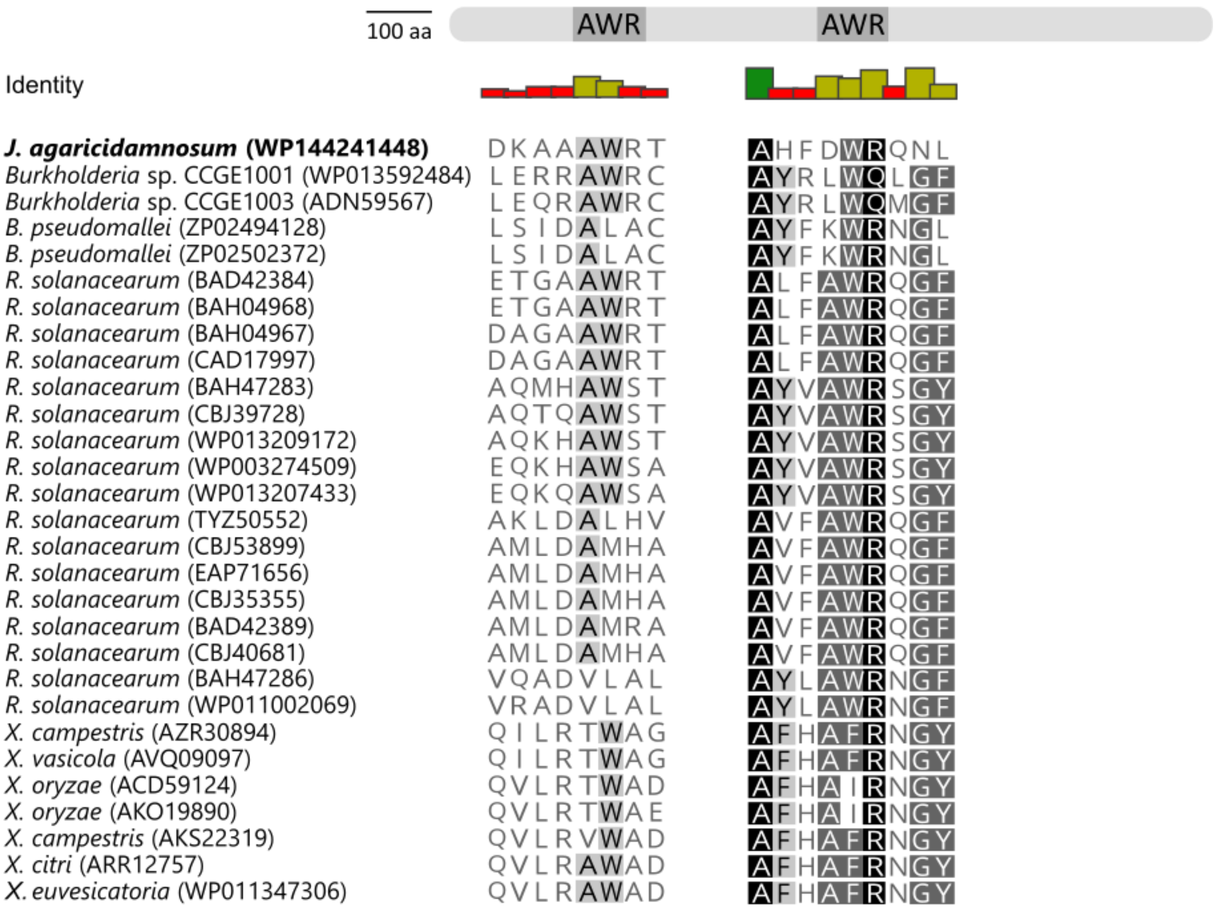

Fig. S4 Sequence alignment of conserved regions in AWR proteins. Shown are the distinctive alanine-tryptophan-arginine triads from the putative T3 AWR effector of *J. agaricidamnosum* and AWR effectors from *Burkholderia* spp., plant pathogenic *R. solanacearum* and *Xanthomonas* spp. The AWR effector of *J. agaricidamnosum* contains an AWR triad at position 321–323 and an aspartate-tryptophan-arginine triad (DWR) at position 454–456. The bar chart above the alignment indicates the overall sequence identity from low (red) to high (green). NCBI accession numbers are displayed in brackets.

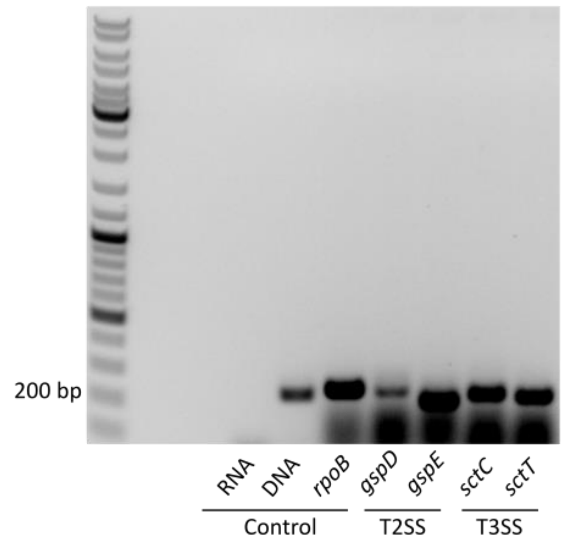

Fig. S5 Gene expression analyses of T2SS- and T3SS-related genes. The gene expression was verified by PCR using complementary DNA (cDNA) as template together with primers for amplifying portions of the appropriate genes. Agarose gel with visualized PCR products indicates that the genes *gspD*, *gspE*, *sctC* and *sctT* are expressed under laboratory conditions. PCR with isolated RNA as template was used as negative control to exclude contaminations with DNA. Genomic DNA served as positive PCR control by using primers to amplify a portion of *gspD*. The amplification of a segment from the housekeeping gene *rpoB* was used as positive control of gene expression. The expected amplicon sizes were 241 bp for *rpoB*, 237 bp for *gspD* (and DNA control), 207 bp for *gspE*, 226 bp for *sctC*, and 223 bp for *sctT*. The GeneRuler 1 kb DNA ladder (Thermo Scientific) was used for sizing.

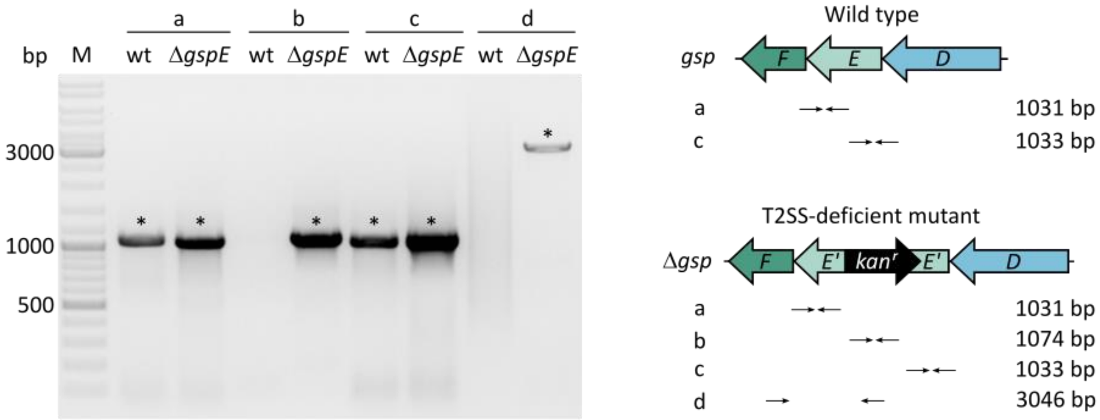

Fig. S6 Verification of the mutant *J. agaricidamnosum* Δ*gspE*. Agarose gel shows that PCR-amplification of product b and d occur only for T2SS-deficient *J. agaricidamnosum* Δ*gspE*, indicating the integration of the kanamycin resistance cassette (*kan*^{r}) into the gene *gspE* by a single-crossover event. Bands corresponding to the expected size are indicated by asterisks (*). M, GeneRuler 1 kb DNA ladder (Thermo Scientific); wt, wild type.

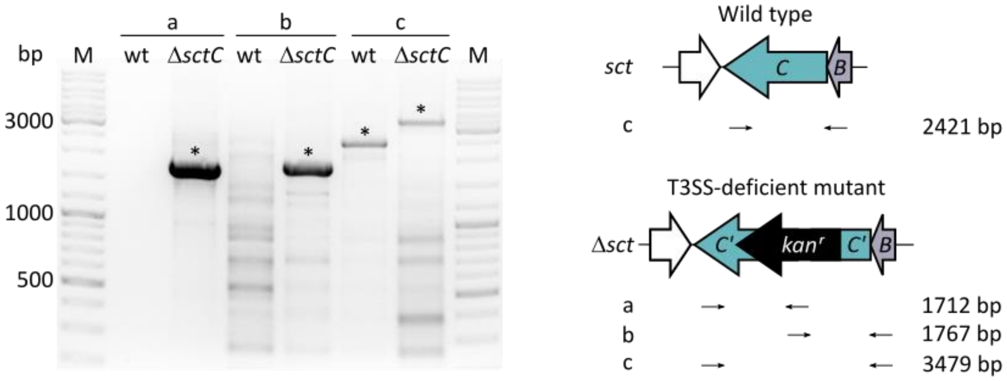

Fig. S7 Verification of the mutant *J. agaricidamnosum* Δ*sctC*. Agarose gel shows that amplicons of product a and b occur only for the T3SS-deficient *J. agaricidamnosum* Δ*sctC*, indicating the integration of the kanamycin resistance cassette (*kan*^{r}) into *sctC* by a double-crossover event. Furthermore, as expected, product c is 3479 bp in the case of the mutant compared to 2421 bp for wild type *J. agaricidamnosum*. Bands corresponding to the expected size are indicated by asterisks (*). M, GeneRuler 1 kb DNA ladder (Thermo Scientific); wt, wild type.

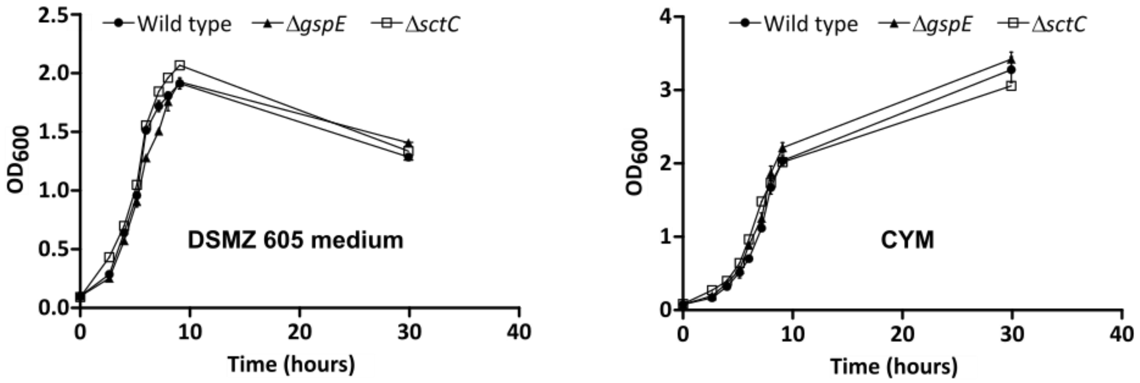

Fig. S8 Growth kinetics of *J. agaricidamnosum*, *J. agaricidamnosum* Δ*gspE* and *J. agaricidamnosum* Δ*sctC*. Cells were cultured in either modified nutrient medium (DSMZ 605 medium) (left) or CYM (right). Data are displayed as means ± SD.

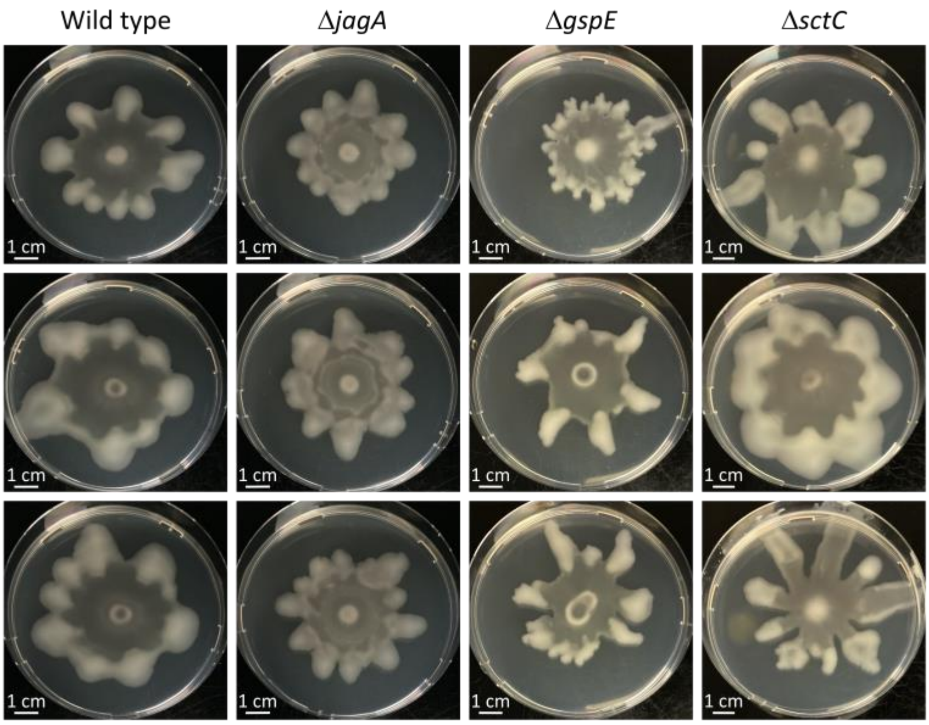

Fig. S9 Swarming assay of *J. agaricidamnosum*, jagaricin-deficient *J. agaricidamnosum* Δ*jagA* and the secretion-deficient *J. agaricidamnosum* Δ*gspE* and *J. agaricidamnosum* Δ*sctC*. The experiments were performed in biological triplicates. Photos were taken from the top of the plate 48 h after inoculation on LB soft agar (0.6 %).

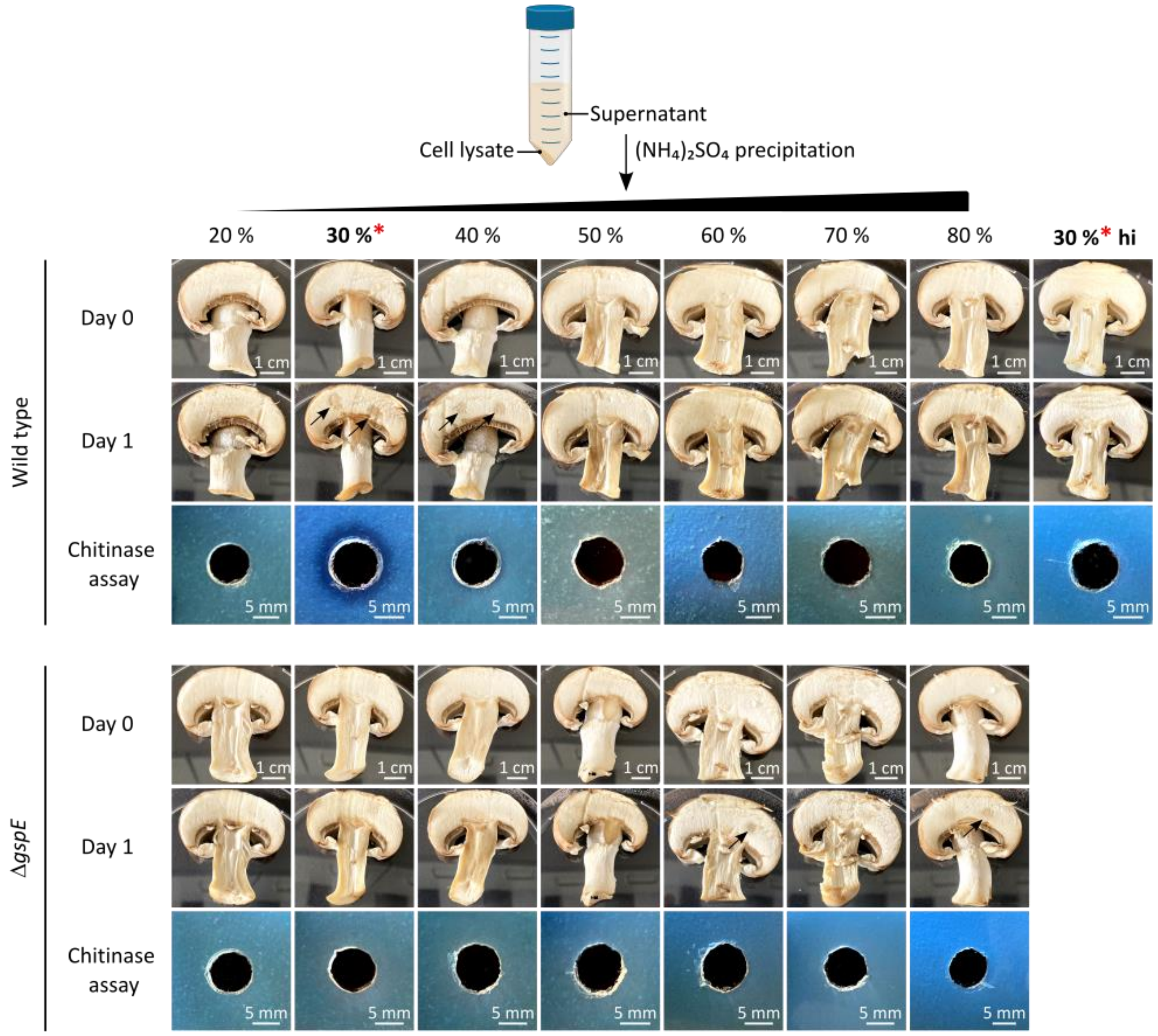

Fig. S10 Bioactivity-guided protein fractionation. Cultures of *J. agaricidamnosum* and *J. agaricidamnosum* Δ*gspE* were incubated in button mushroom-containing medium overnight. Protein fractions were obtained from stepwise $(NH_4)_2SO_4$ precipitation (20 % to 80 % saturation) of sterile-filtered supernatant. Resolubilized protein fractions were tested on two different areas of button mushroom slices and incubated for 1 day. Additionally, the fractions were tested in a calcofluor chitinase assay. The 30 % saturated fraction of wild type *J. agaricidamnosum* supernatant (red asterisk) caused lesions on the button mushroom and halo formation, indicating tissue-degrading activity and chitinase activity, respectively. Arrows point to lesions and browning evoked by protein fractions. The activity was eliminated by heat treatment, and was absent in the corresponding fraction from *J. agaricidamnosum* Δ*gspE.* hi, heat-inactivated.

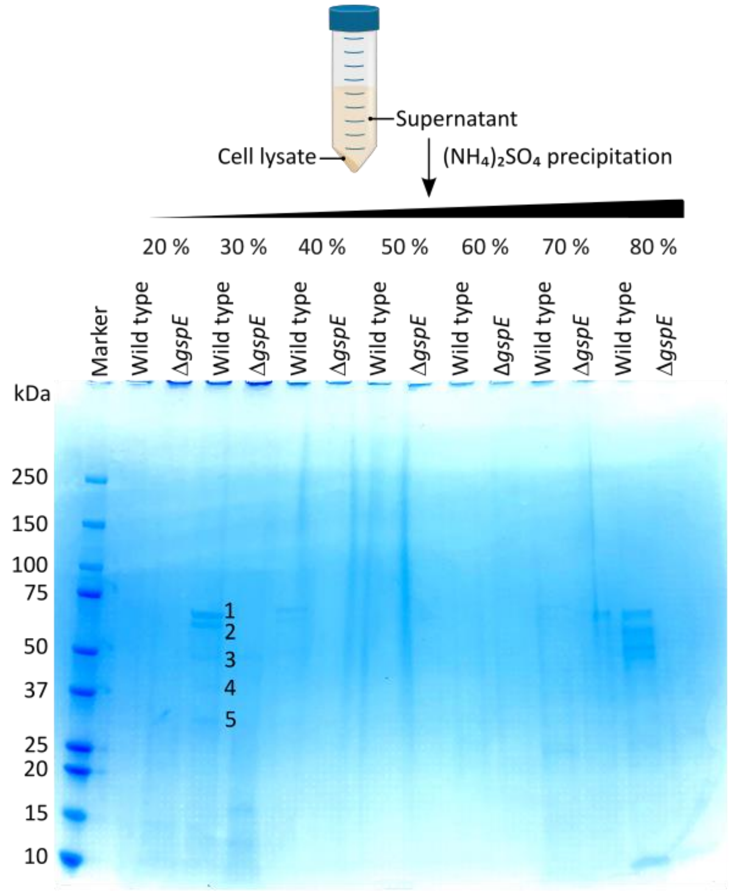

Fig. S11 SDS-PAGE analysis of protein fractions. The bands 1–5 of the bioactive wild type fraction (30 %) were excised from the SDS gel, tryptic digested and analyzed by MALDI-TOF-MS. Marker, Precision Plus Protein Dual Color (Bio-Rad); 1, Ricin-type beta-trefoil lectin domain protein; 2, Glycoside hydrolase family (GH) 18 Chitinase; 3, no matching peptides found; 4, no matching peptides found; 5, GH 19 Chitinase. See Supplementary Figure S11 for bioactivity assays and Figures S12–S16 for MALDI-TOF-MS results.

A

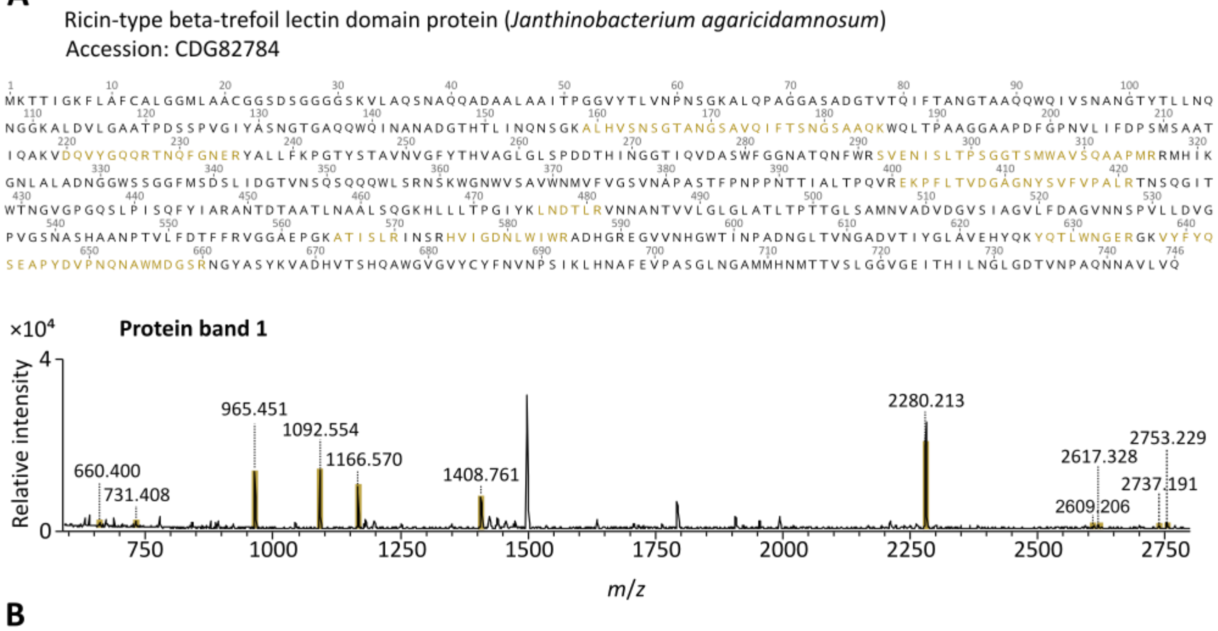

B

Protein band	Peptide sequence	Modification	Missed cleavages	Theoretical. $[M+H]^+$ [Da]	Detected $[M+H]^+$ [Da]
	VYFYQSEAPYDVPNQNAWMDGSR	Oxidation	0	2753.193	2753.229
	VYFYQSEAPYDVPNQNAWMDGSR		0	2737.198	2737.191
	ALHVSNSGTANGSAVQIFTSNGSAAQK		0	2617.296	2617.328
	SVENISLTPSGGTSMWAVSQAAPMR	Oxidation	0	2609.233	2609.206
	EKPFLTVDGAGNYSVFVPALR		0	2280.202	2280.213
1	HVIGDNLWIWR		0	1408.748	1408.761
	YQTLWNGER		0	1166.558	1166.570
	VDQVYGQQR		0	1092.543	1092.554
	TNQFGNER		0	965.443	965.451
	LNDTLR		0	731.404	731.408
	ATISLR		0	660.403	660.400

C

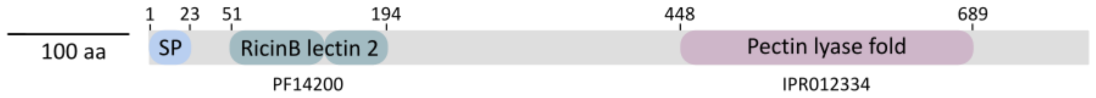

Fig. S12 MALDI-TOF-MS and domain analysis of protein 1. (**A**) Amino acid sequence and MALDI-TOF-MS spectrum of the ricin-type beta-trefoil lectin domain protein (CDG82784) from *J. agaricidamnosum*. The detected masses were searched against masses identified by LC-MS/MS secretome analysis. Matching peptides are highlighted in yellow. (**B**) Detected peptide masses compared to theoretical masses of the *in silico* cleaved protein using the PeptideMass tool on the ExPASy Server (10). (**C**) Schematic illustration of protein domains predicted with InterPro (11). Protein 1 contains a secretion signal peptide (SP) at position 1–23, a ricinB lectin 2 domain (PF14200) at position 51–194, and a pectin lyase fold (IPR012334) at position 448–689.

A

GH18 Chitinase (*Janthinobacterium agaricidamnosum*)
Accession: CDG84085

```
MVLLLASGGLLAACGGDSSNSAAQSKMLAASASAAACSAPWVASTAYTGGATVSYNGVNYKANYWTQGNNPATSNGGPGTGQPWTKVDDCSATPTPTPTPTPTPTPT
PTPTPTPTPTPTPTPTPTPTPNGREVGSYFTQWGVYDRDYEVANIQSSGVAGKLTFINYAFGNIYAKNGGYECGMITKAEPGASNPNAPDAGTGGDAESDYIRTPKR
TVDGQAIPWDAPLSGNFLQFKKLKALNPNLKVLISLGGWSWSKNFSVGSATDALRKQLVRSCIDIYLKGNLPVQGGRGGPGAAKGVFDGIDIDWEYPVGGGQPYNTN
SPADKRNYTLLMAEFRSQLDALGAIDGKRYLLTAAVGAGKDKVDNTEAALYSQYMDWINVMTYDFHGGWENTTNFNAHLYADPNDPSVGVVREYTADKSIQYMIASG
VPRSKLLFGIPFYGRGWTGVAPGPNGNGLYQSATGPAQGKYESGIEDYQILATKPGTRYYHPVTKQLYLYTGAGGQWWSYDDATVIGTKVQYVKDQNLRGAFSWELD
GDPSGTLATEVWKAR
```

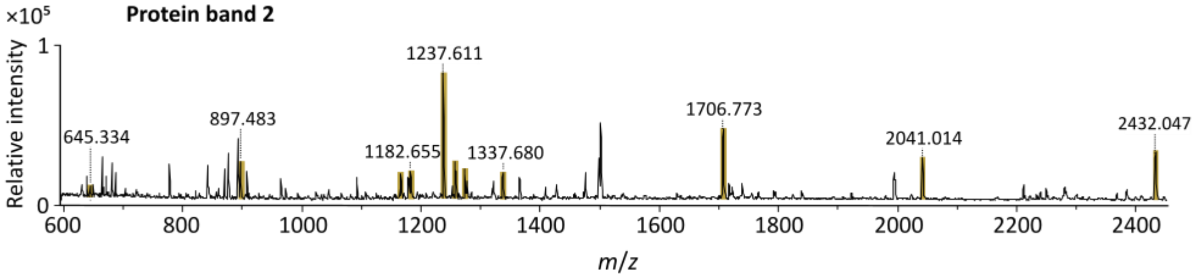

B

Protein band	Peptide sequence	Modification	Missed cleavages	Theoretical. $[M+H]^+$ [Da]	Detected $[M+H]^+$ [Da]
	AEPGASNPNAPDAGTGGDAESDYIR		0	2432.059	2432.047
	YESGIEDYQILATKPGTR		0	2041.023	2041.014
	EVGSYFTQWGVYDR		0	1706.780	1706.773
	SIQYMIASGVPR	Oxidation	0	1337.688	1337.680
	NYTLLMAEFR	Oxidation	0	1273.624	1273.616
2	NYTLLMAEFR		0	1257.629	1257.624
	NFSVGSATDALR		0	1237.617	1237.611
	LLFGIPFYGR		0	1182.666	1182.655
	NGGYECGMITK		0	1172.507	1172.533
	GNLPVQGGR		0	897.490	897.483
	DQNLR		0	645.331	645.334

C

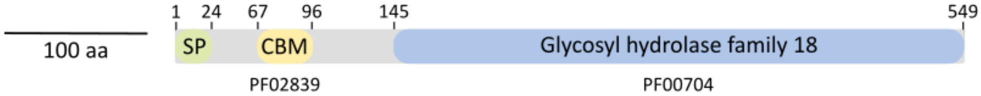

Fig. S13 MALDI-TOF-MS and domain analysis of protein 2. (**A**) Amino acid sequence and MALDI-TOF-MS spectrum of the GH 18 chitinase (CDG84085) from *J. agaricidamnosum*. The detected masses were searched against masses identified by LC-MS/MS secretome analysis. Matching peptides are highlighted in yellow. (**B**) Detected peptide masses compared to theoretical masses of the *in silico* cleaved protein using the PeptideMass tool on the ExPASy Server. (**C**) Schematic illustration of protein domains predicted with InterPro (11). Protein 2 contains a secretion signal peptide (SP) at position 1–24, a carbohydrate binding module (CBM, PF02839) at position 67–96, and a glycosyl hydrolase family 18 domain (PF00704) at position 145–549.

No matching peptides found

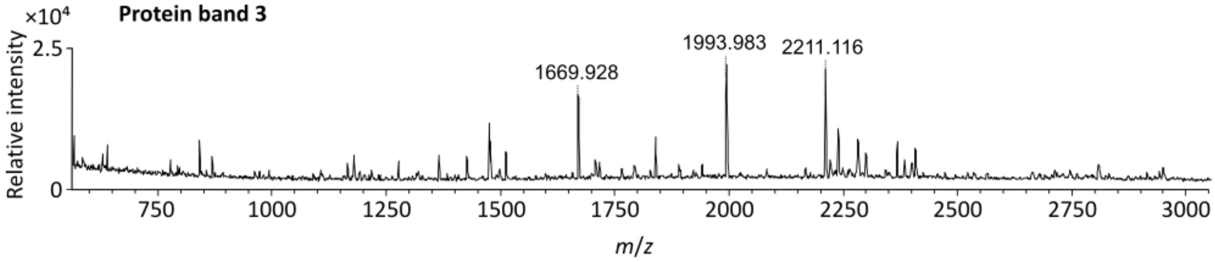

Fig. S14 MALDI-TOF-MS of protein band 3. Peptide masses were searched against masses identified by LC-MS/MS secretome analysis. No matching peptides were found.

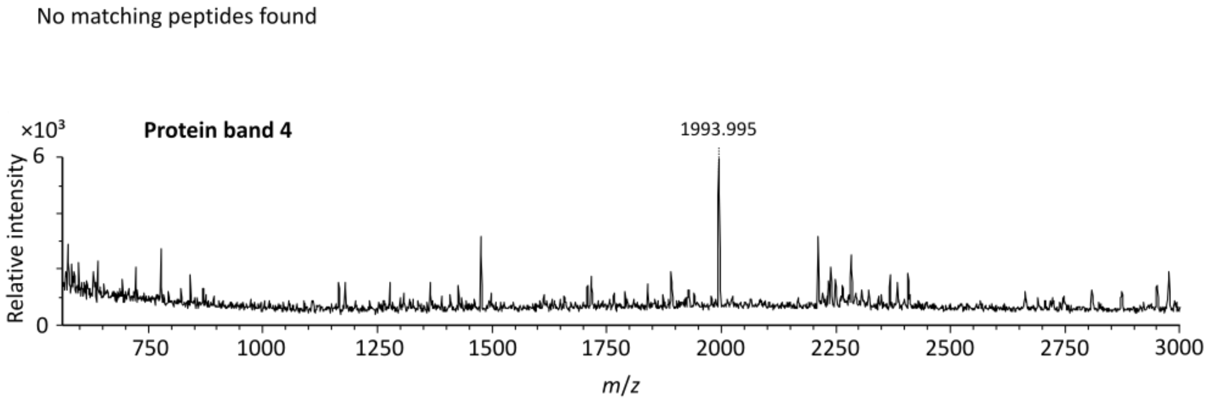

Fig. S15 MALDI-TOF-MS of protein band 4. Peptide masses were searched against masses identified by LC-MS/MS secretome analysis. No matching peptides were found.

A

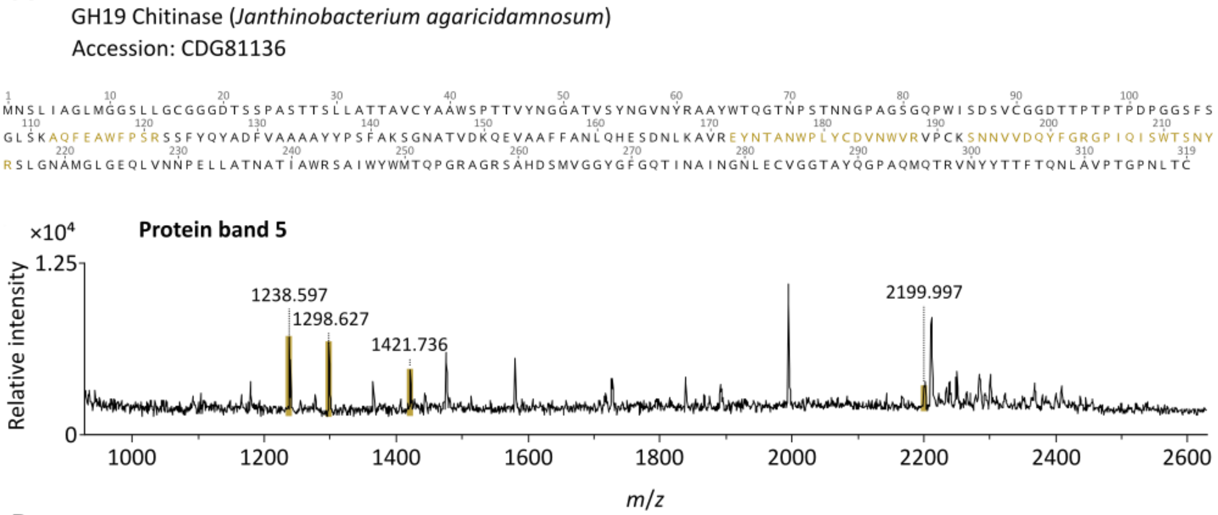

B

Protein band	Peptide sequence	Modification	Missed cleavages	Theoretical. $[M+H]^+$ [Da]	Detected $[M+H]^+$ [Da]
5	EYNTANWPLYCDVNWVR	S-carboxymethylation	0	2199.991	2199.997
	GPIQISWTSNYR		0	1421.717	1421.736
	SNNVVDQYFGR		0	1298.612	1296.627
	AQFEAWFPSR		0	1238.595	1238.597

C

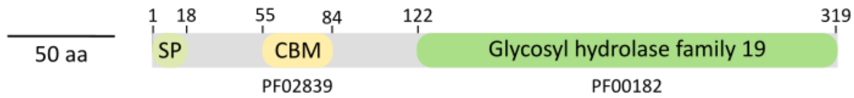

Fig. S16 MALDI-TOF-MS and domain analysis of protein band 5. (**A**) Amino acid sequence and MALDI-TOF-MS spectrum of the GH 19 chitinase (CDG81136) from *J. agaricidamnosum*. The detected masses were searched against masses identified by LC-MS/MS secretome analysis. Matching peptides are highlighted in yellow. (**B**) Detected peptide masses compared to theoretical masses of the *in silico* cleaved protein using the PeptideMass tool on the ExPASy Server. (**C**) Schematic illustration of protein domains predicted with InterPro (11). Protein 5 contains a signal peptide for secretion at position 1–18, a carbohydrate binding module (CBM, PF02839) at position 55–84 and a glycosyl hydrolase family 19 domain (PF00182) at position 122–319.

Table S1. Secretion associated genes and proposed function of encoded proteins

Secretion system	Locus tag	Gene	Proposed function of encoded protein	*E*-value[a]
T1SS	GJA_RS14465	*tolC*	Outer membrane protein	$2.9E^{-32}$
	GJA_RS14470	*abc*	ATP-binding cassette	$1.4E^{-64}$
	GJA_RS14475	*mfp*	Membrane fusion protein	$1.2E^{-29}$
T2SS	GJA_RS23445		Transcription regulator	$6.8E^{-6}$
	GJA_RS23450		Patatin-like phospholipase	$6.0E^{-32}$
	GJA_RS23455	*gspC*	Connecting inner and outer membrane complex	$5.6E^{-15}$
	GJA_RS23460	*gspF*	Inner membrane component, interacts with GspE	$1.5E^{-13}$
	GJA_RS23465	*gspE*	Cytosolic ATPase	$2.2E^{-72}$
	GJA_RS23470	*gspD*	Secretin, forms outer membrane pore	$1.7E^{-70}$
	GJA_RS23475	*gspN*	Anchoring protein, inner membrane platform for pseudopilins	$5.0E^{-164}$
	GJA_RS23480	*gspM*	Anchoring protein, inner membrane platform for pseudopilins	$1.5E^{-10}$
	GJA_RS23485	*gspL*	Anchoring protein, inner membrane platform for pseudopilins	$7.4E^{-24}$
	GJA_RS23490	*gspK*	Minor pseudopilin	$8.9E^{-31}$
	GJA_RS23495	*gspJ*	Minor pseudopilin	$3.7E^{-14}$
	GJA_RS23500	*gspI*	Minor pseudopilin	$2.7E^{-7}$
	GJA_RS23505	*gspH*	Minor pseudopilin	$2.8E^{-14}$
	GJA_RS23510	*gspG*	Major pseudopilin	$8.9E^{-9}$
	GJA_RS23515		Adenylyltransferase	$1.1E^{-32}$
	GJA_RS23520		Peptidase S41	$1.4E^{-48}$
	GJA_RS23525		Murein hydrolase activator	$3.8E^{-34}$
T3SS	GJA_RS07730	*sctV*	Inner membrane machinery	$2.9E^{-161}$
	GJA_RS07735	*sctG*	Chaperone	0.56
	GJA_RS26040		Flagellar biosynthetic protein FlhB	99.0
	GJA_RS07745		Chaperone	62.0
	GJA_RS07750	*sctW*	Chaperone	$5.5E^{-36}$
	GJA_RS07755	*sctD*	Inner membrane ring protein	$6.6E^{-16}$
	GJA_RS07760	*sctN*	Cytosolic ATPase	$1.5E^{-51}$
	GJA_RS07765	*sctO*	Stalk	$7.9E^{-3}$
	GJA_RS26045	*sctP*	Needle length regulator	16.0
	GJA_RS07775	*sctQ*	Dynamic sorting platform	$2.0E^{-12}$
	GJA_RS07780	*sctR*	Inner membrane export apparatus	$1.1E^{-72}$
	GJA_RS07785	*sctS*	Inner membrane export apparatus	$8.7E^{-30}$
	GJA_RS07790	*sctT*	Inner membrane export apparatus	$1.6E^{-46}$
	GJA_RS07795	*sctU*	Inner membrane export apparatus	$1.1E^{-36}$
	GJA_RS07800		Unknown	-
	GJA_RS07805		Chaperone	$8.4E^{-3}$
	GJA_RS07810		GNAT-family acetyltransferase	$1.3E^{-13}$
	GJA_RS27760		Unknown	-
	GJA_RS26050		Transglycosylase SLT	$5.2E^{-12}$
	GJA_RS07825	ORF 20	Putative AWR type III effector[b]	$5.0E^{-32}$
	GJA_RS07835	ORF 21	Putative type III effector[b]	0.95
	GJA_RS07840		Foldase	91.0
	GJA_RS27170	ORF 23	Putative type III effector[b]	$1.0E^{-3}$
	GJA_RS07850		Unknown	-
	GJA_RS07855	ORF 25	Putative type III effector[b]	$4.0E^{-19}$
	GJA_RS07860	ORF 26	Putative type III effector[b]	$4.0E^{-59}$
	GJA_RS07865	ORF 27	Putative type III effector[b]	$1.0E^{-64}$
	GJA_RS07870		Twitching motility pilus retraction ATPase	$1.4E^{-43}$
	GJA_RS27175		DNA-repair protein	94.0
	GJA_RS07880		RNA polymerase sigma-H factor	$1.8E^{-25}$
	GJA_RS07885	*sctI*	Inner rod	$1.2E^{-19}$
	GJA_RS07890	*sctJ*	Inner membrane ring protein	$5.5E^{-41}$

	GJA_RS26055	*sctK*	Dynamic sorting platform	4.8E^{-2}
	GJA_RS07900	*sctL*	ATPase regulator, dynamic sorting platform	4.7E^{-13}
	GJA_RS07905		Transcription elongation factor	95.0
	GJA_RS27180		Unknown	-
	GJA_RS07910		DNA-binding response regulator	4.8E^{-30}
	GJA_RS07915	*sctC*	Outer membrane secretin ring	4.7E^{-13}
	GJA_RS27765	*sctB*	Translocon, Chaperone	1.8E^{-7}
	GJA_RS11900	*tssM*	Inner membrane anchoring protein, connects TssJ to TssL	8.5E^{-109}
	GJA_RS11905	*tagF*	Accessory protein, T6SS negative regulator	9.0E^{-170}
	GJA_RS11910	*ompA*	Outer membrane protein	4.4E^{-6}
	GJA_RS11915	*tssA*	Baseplate assembly	2.1E^{-37}
	GJA_RS11920		Unknown	-
	GJA_RS11925		Unknown	-
	GJA_RS11930	*tssH*	AAA+ ATPase, disassembles contracted TssB–TssC	1.4E^{-52}
T6SS_1	GJA_RS11935	*tssG*	Cytosolic baseplate	2.2E^{-52}
	GJA_RS11940	*tssF*	Cytosolic baseplate	5.1E^{-94}
	GJA_RS11945	*tssE*	Cytosolic baseplate	7.4E^{-18}
	GJA_RS11950	*tssD*	Secreted tube	2.0E^{-33}
	GJA_RS11955	*tssC*	Contractile tail sheath	4.4E^{-96}
	GJA_RS11960	*tssB*	Contractile tail sheath	9.9E^{-29}
	GJA_RS11965	-	Lipoprotein, serin/threonin phosphatase	1.2E^{-10}
	GJA_RS11970	*tssK*	Cytosolic baseplate	1.2E^{-78}
	GJA_RS11975	*tssL*	Inner membrane anchoring protein	1.3E^{-40}
	GJA_RS26685	*tssI/vgrG*	Puncture tip	1.8E^{-45}
	GJA_RS15800		*S*-adenosylmethionine dependent 3-amino-3-carboxypropyl transferase	5.8E^{-21}
	GJA_RS15805		Methyl-accepting chemotaxis protein	9.3E^{-35}
	GJA_RS15810		Outer membrane lipoprotein	1.4E^{-26}
	GJA_RS15815	*tssD*	Secreted tube	5.2E^{-34}
	GJA_RS15820		Serin/threonin phosphatase	6.0E^{-12}
T6SS_2	GJA_RS15825	*tssL*	Inner membrane anchoring protein	5.1E^{-41}
	GJA_RS15830	*ompA*	Outer membrane protein	4.3E^{-6}
	GJA_RS15835	*tagF*	Accessory protein, T6SS negative regulator	1.0E^{-142}
	GJA_RS15840	*tssM*	Inner membrane anchoring protein, connects TssJ to TssL	2.8E^{-110}
	GJA_RS26810		L-alanyl-D-glutamate peptidase	2.3E^{-17}
	GJA_RS25040	*tssA*	Baseplate assembly	5.4E^{-37}
	GJA_RS25035	*tssB*	Contractile tail sheath	1.2E^{-28}
	GJA_RS25030	*tssC*	Contractile tail sheath	3.6E^{-96}
	GJA_RS25025	*tssD*	Secreted tube	2.0E^{-32}
	GJA_RS25020	*tssE*	Cytosolic baseplate	6.6E^{-17}
	GJA_RS25015	*tssF*	Cytosolic baseplate	1.8E^{-99}
	GJA_RS25010	*tssG*	Cytosolic baseplate	1.4E^{-51}
	GJA_RS25005	*tssH*	AAA+ ATPase, disassembles contracted TssB-TssC	1.2E^{-50}
T6SS_3	GJA_RS25000	*vgrG/tssI*	Puncture tip	3.9E^{-71}
	GJA_RS24995	*tagH*	Accessory protein, Serine/threonine kinase, T6SS regulator	2.0E^{-86}
	GJA_RS24990	*tssK*	Cytosolic baseplate	3.2E^{-79}
	GJA_RS24985	*tssL*	Inner membrane anchoring protein	6.6E^{-42}
	GJA_RS24980	*tssM*	Inner membrane anchoring protein, connects TssJ to TssL	2.6E^{-106}
	GJA_RS24975		Serine/threonine phosphatase, T6SS regulator (PppA-like)	2.1E^{-21}
	GJA_RS24970	*tagF*	Accessory protein, T6SS negative regulator	1.0E^{-74}

[a]*E*-values of the proposed proteins were obtained using HHPred or NCBI BLAST (6, 7).
[b]Type III secretion signals were predicted using the Effective T3 prediction tool (8).

Table S2. Potentially relevant T3 effector proteins encoded within the T3SS gene cluster

Locus tag	ORF	Proposed function of encoded protein[a]	Similar protein sequences (Organism)	Percent identity[b]
GJA_RS07825	20	Type III effector	AWR/RipA5-effector family protein (*R. solanacearum*)	24
GJA_RS07835	21	Type III effector	Hypothetical protein (*B. vietnamiensis*)	47
GJA_RS27170	23	Type III effector	Type III effector (*X. campestris* pv. *incanae*)	28
GJA_RS07855	25	Type III effector	Type III effector (*X. euvesicatoria*)	37
GJA_RS07860	26	Type III effector	Type III effector HopAS1 (*P. syringae* pv. *philadelphi*)	27
GJA_RS07865	27	Type III effector	Type III effector (*P. viridiflava* ICMP 13104)	28

[a]Type III secretion signals were predicted using the Effective T3 prediction tool (8).
[b]Percent identities of similar protein sequences were obtained using NCBI BLAST (7).

Table S3. Proteins identified by bioactivity-guided fractionation and MALDI-TOF-MS[a]

Protein band	NCBI accession #	Protein identification	Mass (kDa)	SP[b]	Identified peptide sequences
**1**	CDG82784	Ricin-type beta-trefoil lectin domain protein	77.8	yes	VYFYQSEAPYDVPNQNAWMDGSR<br>VYFYQSEAPYDVPNQNAWMDGSR<br>ALHVSNSGTANGSAVQIFTSNGSAAQK<br>SVENISLTPSGGTSMWAVSQAAPMR<br>EKPFLTVDGAGNYSVFVPALR<br>HVIGDNLWIWR<br>YQTLWNGER<br>VDQVYGQQR<br>TNQFGNER<br>LNDTLR<br>ATISLR
**2**	CDG84085	Chitinase (Glycoside hydrolase family 18)	59.8	yes	AEPGASNPNAPDAGTGGDAESDYIR<br>YESGIEDYQILATKPGTR<br>EVGSYFTQWGVYDR<br>SIQYMIASGVPR<br>NYTLLMAEFR<br>NYTLLMAEFR<br>NFSVGSATDALR<br>LLFGIPFYGR<br>NGGYECGMITK<br>GNLPVQGGR<br>DQNLR
**5**	CDG81136	Chitinase (Glycoside hydrolase family 19)	29.0	yes	EYNTANWPLYCDVNWVR<br>GPIQISWTSNYR<br>SNNVVDQYFGR<br>AQFEAWFPSR

[a]Peptides were manually searched against proteins identified by LC-MS/MS secretome analysis and *in silico* cleaved putative exoproteins of *J. agaricidamnosum* DSM9628 using the PeptideMass tool on the ExPASy Server (10).

[b]Secretion signal-containing peptide (SP). SignalP 6.0 was used to predict whether the peptides would be secreted.

Table S4. First ten results returned in a HHpred search of the RICIN domain-containing protein (CDG82784)

No.	PDBe	Name	Organism	*E*-value	Identity
1	4TZ1	Exo-1,3-β-glucanase	*Streptomyces* sp. SirexAA-E	$3.4E^{-91}$	54 %
2	5M5Z	β-1-3-glucanase	*Chaetomium thermophilum*	$1.5E^{-50}$	21 %
3	3EQN	β-1-3-glucanase	*Phanerochaete chrysosporium*	$2.3E^{-47}$	21 %
4	2F2F	Cytolethal distending toxin	*Actinobacillus actinomycetemcomitans*	$4.8E^{-8}$	14 %
5	4PC4	30-kDa lipoprotein	*Bombyx mori*	$8.1E^{-7}$	14 %
6	7QE4	β-trefoil lectin	*Salpingoeca rosetta*	$1.0E^{-6}$	17 %
7	5XG5	β-trefoil lectin	Synthetic construct	$2.6E^{-5}$	20 %
8	4A7K	Aldos-2-ulose dehydratase	*Phanerochaete chrysosporium*	$3.3E^{-5}$	18 %
9	4U49	Pectate lyase Pel3	*Pectobacterium carotovorum*	$1.6E^{-5}$	11 %
10	8BAD	Binary toxin A-like protein	*Bacillus thuringiensis*	$2.2E^{-4}$	22 %

Table S5. Strains used in this study

Strains	Relevant genotype and descriptions	Source
Janthinobacterium agaricidamnosum	Wild type (DSM 9628)	DSMZ
Janthinobacterium agaricidamnosum Δ*jag*	Inactivated jagaricin biosynthesis gene cluster	(12)
Janthinobacterium agaricidamnosum Δ*gspE*	Inactivated T2SS gene cluster	This study
Janthinobacterium agaricidamnosum Δ*sctC*	Inactivated T3SS gene cluster	This study
Escherichia coli HST08 Stellar	Chemically competent cells	Takara Bio
Escherichia coli ER2925	Methyltransferase deficient electrocompetent strain, *dam*$^{-}$/*dcm*$^{-}$	NEB/Laboratory strain

Table S6. Oligonucleotide primers used in this study

**Name**	**Sequence 5'→3'**	**Application**
RT_*sctC*_fw	CCTGGCCAATATTTCAGTCG	*sctC* expression analysis
RT_*sctC*_rv	GTTCAGTTTTTCGGCATGGT	
RT_*sctT*_fw	GCTTTTCCGAATTTGTCTGG	*sctT* expression analysis
RT_*sctT*_rv	TCGAGGATGTGGGAAAAGTC	
RT_*gspE*_fw	GTCGAGAACACCGTCGAAGT	*gspE* expression analysis
RT_*gspE*_rv	CAGCTTGGTCAAGTCCAGGT	
RT_*gspD*_fw	TTTCCTGCATTCTCCTCGTT	*gspD* expression analysis
RT_*gspD*_rv	GATCGATTTTTCGGAGACCA	
RT_*rpoB*_fw	GGTCGAAGGTTCCAAGATCA	*rpoB* expression analysis
RT_*rpoB*_rv	GTGTTCGAGGAAGGGAATCA	
*gspE*_hr1_fw	GAATTCCTGCAGCCCCCAGACCCGCACGCGCAAGAAGACC	pKO*gspE* cloning
*gspE*_hr1_rv	TCTTCTGAGCAGCATGTCGCCGCTCATGCCG	
*gspE*_hr2_fw	ATTCATCCCGCAGTTCGATGCGCTGATCAAGC	
*gspE*_hr2_rv	ACTAGTGGATCCCCCGCCGAGAACGCCTGTTCCAGCG	
gspE_kan^{r}_fw	CATGCTGCTCAGAAGAACTCGTCAAGAAGGCGATAG	
gspE_kan^{r}_rv	GAACTGCGGGATGAATGTCAGCTACTGGGCTATCTGG	
*sctC*_hr1_fw	GAATTCCTGCAGCCCCCCAATCGCCACGGCAACGCCAGG	pKO*sctC* cloning
*sctC*_hr1_rv	TCTTCTGAGCTTCGATTTCAATCTGGCGCGGTTCGACATCG	
*sctC*_hr2_fw	AATCGAAGCTCAGAAGAACTCGTCAAGAAGGCGATAG	
*sctC*_hr2_rv	TGATCAGCGGATGAATGTCAGCTACTGGGCTATCTGG	
sctC_kan^{r}_fw	ATTCATCCGCTGATCATCGATATCGACCGCGACAAGCTGG	
sctC_kan^{r}_rv	ACTAGTGGATCCCCCGCAAGGTCACCATCCGCACCGCCTACAGC	
M13_fw	AGGGTTTTCCCAGTCACGACGTT	pKO*gspE* and pKO*sctC* insert sequencing
M13_rv	GAGCGGATAACAATTTCACACAGG	
*gspE*_hr1-*kan*^{r}_fw	CCGTGAGCACACCTAAATTTCCTGC	Δ*gspE* verification
*gspE*_hr1-*kan*^{r}_rv	CGTGATATTGCTGAAGAGCTTGGCG	
gspE_kan^{r}-hr2_fw	CGCCAAGCTCTTCAGCAATATCACG	
gspE_kan^{r}-hr2_rv	GCTGCACTTCCTTGACCGTCACG	
*sctC*_hr1-*kan*^{r}_fw	GGAAACCTTGTTGAACCAGGTCG	Δ*sctC* verification
*sctC*_hr1-*kan*^{r}_rv	CTGTCCGGTGCCCTGAATGAAC	
*sctC*_hr2-*kan*^{r}_fw	GTTCATTCAGGGCACCGGACAG	
*sctC*_hr2-*kan*^{r}_rv	GCATATGCATTTCGAGCCGGTGG	

Table S7. Plasmids used in this study

Plasmid	Description	Source
pGL42a	Cloning vector	(13)
pK19	Contains kanamycin resistance cassette (kan^r)	(14)
pKO*gspE*	pGL42a with homology arms and kan^r for *gspE* (GJA_RS23465) disruption	This study
pKO*sctC*	pGL42a with homology arms and kan^r for *sctC* (GJA_RS07915) disruption	This study

Table S8. Quantification of mushroom (*A. bisporus*) degradation caused by *J. agaricidamnosum* strains. The listed data derived from four independent experiments with six replicates each. Each replicate consisted of four sliced and weighed mushrooms (approx. 0.5 cm thickness) that were treated with *J. agaricidamnosum* (wild type), *J. agaricidamnosum* Δ*gspE*, or *J. agaricidamnosum* Δ*sctC*. Slices were spotted with culture medium to give negative (untreated) controls. After inoculation the slices were incubated at room temperature for 6 days, then the mushroom tissue was re-weighed. The mushroom degradation is calculated in percent as follows: % weight loss = 100 − (final weight / starting weight × 100). Expt., Experiment.

Expt.	Mushroom + wild type (% weight loss)						Mushroom + Δ*gspE* (% weight loss)					
1	77.12	76.63	63.48	69.35	74.11	91.03	11.28	15.67	15.94	9.95	13.94	12.94
2	45.36	65.17	30.31	20.63	30.74	44.49	16.80	19.79	18.86	21.30	17.59	16.71
3	93.03	53.49	50.07	69.44	68.60	64.73	36.48	13.68	13.02	25.37	40.73	19.17
4	71.89	72.82	71.10	68.29	74.39	80.26	8.80	10.96	9.57	10.32	9.19	10.28

Expt.	Mushroom + Δ*sctC* (% weight loss)						Mushroom + culture medium (% weight loss)					
1	15.89	23.82	14.36	17.09	16.29	13.28	7.61	9.42	9.76	7.31	9.22	9.66
2	21.24	17.12	20.43	19.94	23.03	15.31	14.92	12.10	9.81	9.97	6.51	14.91
3	14.55	15.76	13.94	12.05	14.26	17.20	10.90	10.38	8.78	8.67	11.08	10.06
4	21.80	14.66	20.32	16.09	17.43	17.63	7.64	5.89	6.45	6.84	7.35	9.50

Table S9. Statistical analysis of mushroom degradation caused by *J. agaricidamnosum* strains. (**A**) Two-way analysis of variance (ANOVA) and (**B**) Bonferroni's means comparison test for mushroom degradation induced by *J. agaricidamnosum* (wild type), *J. agaricidamnosum* Δ*gspE*, or *J. agaricidamnosum* Δ*sctC*. Uninoculated slices spotted with culture medium served as negative controls. *P*-values < 0.05 were considered statistically significant. DF, Degrees of freedom; Expt., Experiment; Mean Diff., Mean difference; MS, Mean square; SS, Sum of squares.

A

**ANOVA Table**	**DF**	**SS**	**MS**	**F (DFn, DFd)**	***P*-value**
Treatment (between columns)	3	44504	14835	F (3, 80) = 306.7	*P* < 0.0001
Experiment (between rows)	3	693.3	231.1	F (3, 80) = 4.78	*P* = 0.0041
Residual	80	1870	48.37		

B

**Strain comparison**	**Expt.**	**Mean Wild type**	**Mean Δ*gspE***	**Mean Diff.**	**95 % CI of Diff.**	**Summary**	***P*-value**
	1	75.29	13.29	−62.00	−74.77 to −49.22	***	*P* < 0.001
Wild type vs	2	39.45	18.51	−20.94	−33.72 to −8.17	***	*P* < 0.001
Δ*gspE*	3	66.56	24.74	−41.82	−54.60 to −29.04	***	*P* < 0.001
	4	73.13	9.85	−63.27	−76.05 to −50.50	***	*P* < 0.001

**Strain comparison**	**Expt.**	**Mean Wild type**	**Mean Δ*sctC***	**Mean Diff.**	**95 % CI of Diff.**	**Summary**	***P*-value**
	1	75.29	16.79	−58.50	−71.28 to −45.72	***	*P* < 0.001
Wild type vs	2	39.45	19.51	−19.94	−32.71 to −7.16	***	*P* < 0.001
Δ*sctC*	3	66.56	14.63	−51.93	−64.71 to −39.16	***	*P* < 0.001
	4	73.13	17.99	−55.14	−67.91 to −42.36	***	*P* < 0.001

**Strain comparison**	**Expt.**	**Mean Wild type**	**Mean control**	**Mean Diff.**	**95 % CI of Diff.**	**Summary**	***P*-value**
	1	75.29	8.83	−66.46	−79.23 to −53.68	***	*P* < 0.001
Wild type vs	2	39.45	11.37	−28.08	−40.86 to −15.30	***	*P* < 0.001
control	3	66.56	9.98	−56.58	−69.36 to −43.81	***	*P* < 0.001
	4	73.13	7.28	−65.84	−78.62 to −53.07	***	*P* < 0.001

**Strain comparison**	**Expt.**	**Mean Δ*gspE***	**Mean Δ*sctC***	**Mean Diff.**	**95 % CI of Diff.**	**Summary**	***P*-value**
	1	13.29	16.79	3.50	−9.28 to 16.28	ns	*P* > 0.05
Δ*gspE* vs	2	18.51	19.51	1.00	−11.77 to 13.78	ns	*P* > 0.05
Δ*sctC*	3	24.74	14.63	−10.11	−22.89 to 2.66	ns	*P* > 0.05
	4	9.852	17.99	8.14	−4.64 to 20.91	ns	*P* > 0.05

**Strain comparison**	**Expt.**	**Mean Δ*gspE***	**Mean control**	**Mean Diff.**	**95 % CI of Diff.**	**Summary**	***P*-value**
	1	13.29	8.83	−4.46	−17.24 to 8.32	ns	*P* > 0.05
Δ*gspE* vs	2	18.51	11.37	−7.14	−19.92 to 5.64	ns	*P* > 0.05
control	3	24.74	9.98	−14.76	−27.54 to −1.99	**	*P* < 0.01
	4	9.852	7.28	−2.57	−15.35 to 10.21	ns	*P* > 0.05

**Strain comparison**	**Expt.**	**Mean Δ*sctC***	**Mean control**	**Mean Diff.**	**95 % CI of Diff.**	**Summary**	***P*-value**
	1	16.79	8.83	−7.96	−20.73 to 4.82	ns	*P* > 0.05
	2	19.51	11.37	−8.14	−20.92 to 4.63	ns	*P* > 0.05

Δ*sctC* vs	3	14.63	9.98	−4.65	−17.43 to 8.13	ns	$P > 0.05$
control	4	17.99	7.28	−10.71	−23.48 to 2.07	*	$P < 0.05$

References

1. Sievers F, Higgins DG. 2018. Clustal Omega for making accurate alignments of many protein sequences. *Protein Sci* 27:135–145.
2. Kumar S, Stecher G, Tamura K. 2016. MEGA7: molecular evolutionary genetics analysis version 7.0 for bigger datasets. *Mol Biol Evol* 33:1870–4.
3. Saitou N, Nei M. 1987. The neighbor-joining method: a new method for reconstructing phylogenetic trees. *Mol Biol Evol* 4:406–425.
4. Felsenstein J. 1985. Confidence limits on phylogenies: an approach using the bootstrap. *Evolution* 39:783–791.
5. Zuckerkandl E, Pauling L. 1965. Evolutionary divergence and convergence in proteins, p 97–166. *In* Bryson V, Vogel HJ (ed), *Evolving Genes and Proteins* doi:10.1016/B978-1-4832-2734-4.50017-6. Academic Press.
6. Zimmermann L, Stephens A, Nam S-Z, Rau D, Kübler J, Lozajic M, Gabler F, Söding J, Lupas AN, Alva V. 2018. A completely reimplemented MPI bioinformatics toolkit with a new HHpred server at its core. *J Mol Biol* 430:2237–2243.
7. Johnson M, Zaretskaya I, Raytselis Y, Merezhuk Y, McGinnis S, Madden TL. 2008. NCBI BLAST: a better web interface. *Nucleic Acids Res* 36:W5–9.
8. Arnold R, Brandmaier S, Kleine F, Tischler P, Heinz E, Behrens S, Niinikoski A, Mewes H-W, Horn M, Rattei T. 2009. Sequence-based prediction of type III secreted proteins. *PLOS Pathog* 5:e1000376.
9. Solé M, Popa C, Mith O, Sohn KH, Jones JDG, Deslandes L, Valls M. 2012. The *awr* gene family encodes a novel class of *Ralstonia solanacearum* type III effectors displaying virulence and avirulence activities. *Mol Plant Microbe Interact* 25:941–953.
10. Wilkins MR, Lindskog I, Gasteiger E, Bairoch A, Sanchez JC, Hochstrasser DF, Appel RD. 1997. Detailed peptide characterization using PEPTIDEMASS – a World-Wide-Web-accessible tool. *Electrophoresis* 18:403–8.
11. Blum M, Chang H-Y, Chuguransky S, Grego T, Kandasaamy S, Mitchell A, Nuka G, Paysan-Lafosse T, Qureshi M, Raj S, Richardson L, Salazar GA, Williams L, Bork P, Bridge A, Gough J, Haft DH, Letunic I, Marchler-Bauer A, Mi H, Natale DA, Necci M, Orengo CA, Pandurangan AP, Rivoire C, Sigrist CJA, Sillitoe I, Thanki N, Thomas PD, Tosatto SCE, Wu CH, Bateman A, Finn RD. 2020. The InterPro protein families and domains database: 20 years on. *Nucleic Acids Res* 49:D344–D354.
12. Graupner K, Scherlach K, Bretschneider T, Lackner G, Roth M, Gross H, Hertweck C. 2012. Imaging mass spectrometry and genome mining reveal highly antifungal virulence factor of mushroom soft rot pathogen. *Angew Chem Int Ed* 51:13173–13177.
13. Lackner G, Moebius N, Hertweck C. 2011. Endofungal bacterium controls its host by an hrp type III secretion system. *ISME J* 5:252–61.
14. Pridmore RD. 1987. New and versatile cloning vectors with kanamycin-resistance marker. *Gene* 56:309–312.
